# The m^6^A reader YTHDF1 attenuates fulminant hepatitis via MFG-E8 translation in an m^6^A dependent manner

**DOI:** 10.7150/ijbs.84768

**Published:** 2023-07-31

**Authors:** Meng-Yun Ke, Yi Fang, Hui Cai, Jian-Wen Lu, Lin Yang, Yue Wang, Rong-Qian Wu, Xu-Feng Zhang, Yi Lv, Jian Dong

**Affiliations:** 1 Department of Vascular Surgery, The First Affiliated Hospital of Xi'an Jiaotong University, Xi'an 710061, Shaanxi Province, China.; 2 National Local Joint Engineering Research Center for Precision Surgery & Regenerative Medicine, Shaanxi Provincial Center for Regenerative Medicine and Surgical Engineering, The First Affiliated Hospital of Xi'an Jiaotong University, 277 West Yanta Road, Xi'an 710061, Shaanxi Province, China.; 3 Institute of Advanced Surgical Technology and Engineering, The First Affiliated Hospital of Xi'an Jiaotong University, 277 West Yanta Road, Xi'an 710061, Shaanxi Province, China.

**Keywords:** YTHDF1, Acute liver failure, m^6^A, MFG-E8, Mitochondria

## Abstract

**Background and Aims:** N6-methyladenosine (m^6^A) is the most common post-transcriptional modification of RNA in eukaryotes, which has been demonstrated to play important roles in various biological processes. However, its roles in fulminant hepatitis remain largely unknown. In the current study, YTHDF1 expression was found to be significantly downregulated in the livers among patients, as well as murine models with fulminant hepatitis versus normal controls. Thus, we hypothesized that YTHDF1 protects against fulminant hepatitis and investigated the underlying molecular mechanisms.

**Methods:** Fulminant hepatitis was induced by D-GalN/LPS in conventional YTHDF1 knockout (YTHDF1^-/-^) mice, hepatocyte-specific YTHDF1 overexpression (AAV8- YTHDF1) mice, and corresponding control mice. Primary hepatocytes were cultured and subjected to LPS insult *in vitro*. Hepatic histology, cell death, oxidative stress and mitochondrial function were examined to assess liver damage. The molecular mechanisms of YTHDF1 function were explored using multi-omics analysis.

**Results:** Ablation of YTHDF1 exacerbated hepatic apoptosis and reactive oxygen species (ROS) production and increased the number of aberrant mitochondria, while YTHDF1 overexpression resulted in the opposite effects. Multiomics analysis identified MFG-E8 as the direct target of YTHDF1. YTHDF1 augmented the translation of MFG-E8 in an m^6^A-dependent manner without effect on its mRNA expression, thereby restoring mitochondrial function. Additionally, administration of MFG-E8 almost completely reversed the YTHDF1 deficiency-mediated exacerbation of liver injury.

**Conclusions:** The current study suggested that the m^6^A reader YTHDF1 alleviates cell death, enhances antioxidant capacity and restores mitochondrial function in fulminant hepatitis by promoting MFG-E8 protein translation in an m^6^A-dependent manner.

## Introduction

Fulminant hepatitis is characterized by acute liver failure (ALF), and can result in encephalopathy, jaundice, and severe coagulopathy with a very high mortality rate but limited treatment options^1^, ^2^. The main causes of ALF are antigen-induced infections, endotoxemia and drug overdose^3^, ^4^. The pathogenic mechanism of ALF has been widely investigated, mitochondrial oxidative stress, a major driver of hepatocyte apoptosis, has received close attention in recent years 5. However, the mechanisms remain elusive. Accordingly, there are still no effective pharmacological treatments approved for use in the clinic.

N6-Methyladenosine (m^6^A), the most abundant internal modification of eukaryotic mRNA^6^, has been reported to participate in cell apoptosis^7^, ^8^, reactive oxygen species^9^ and mitochondrial dysfunction^10^, ^11^, which play vital roles in the pathogenesis of ALF. In eukaryotes, the m^6^A modification is initiated by the m^6^A methyltransferase complex (writers), which is composed by METTL3/14^12^-^14^, WTAP, and RBM15/15B, reversed by demethylases (FTO and ALKBH5; named erasers), and identified by m^6^A binding proteins (YTHDF1/2/3^15^, ^16^, YTHDC1/2^17^-^19^, IGF2BP1/2/3^20^, and HNRNPA2B1; termed “readers^21^”). Accumulating evidence has revealed that abnormal expression of m^6^A regulators can affect diverse biological processes, including various liver diseases 22-25. Whether m^6^A modifications play key roles in the progression of ALF is unknown.

In this study, we performed unbiased assessment of the aberrant expression of m^6^A-associated genes during the pathogenesis of ALF and observed that the expression YTHDF1, an important reader of m^6^A modification, exhibited the most significant decrease in ALF samples. Further deep investigation revealed that YTHDF1 strongly protects against liver damage by maintaining mitochondrial function in a milk fat globule EGF factor 8 (MFG-E8)-dependent manner. Thus, our data demonstrate the critical protective roles of YTHDF1 in ALF, indicating the importance of the homeostasis of m^6^A modification for the pathogenesis and treatment of ALF.

## Results

### YTHDF1 expression was downregulated in ALF

**Figure 1:**
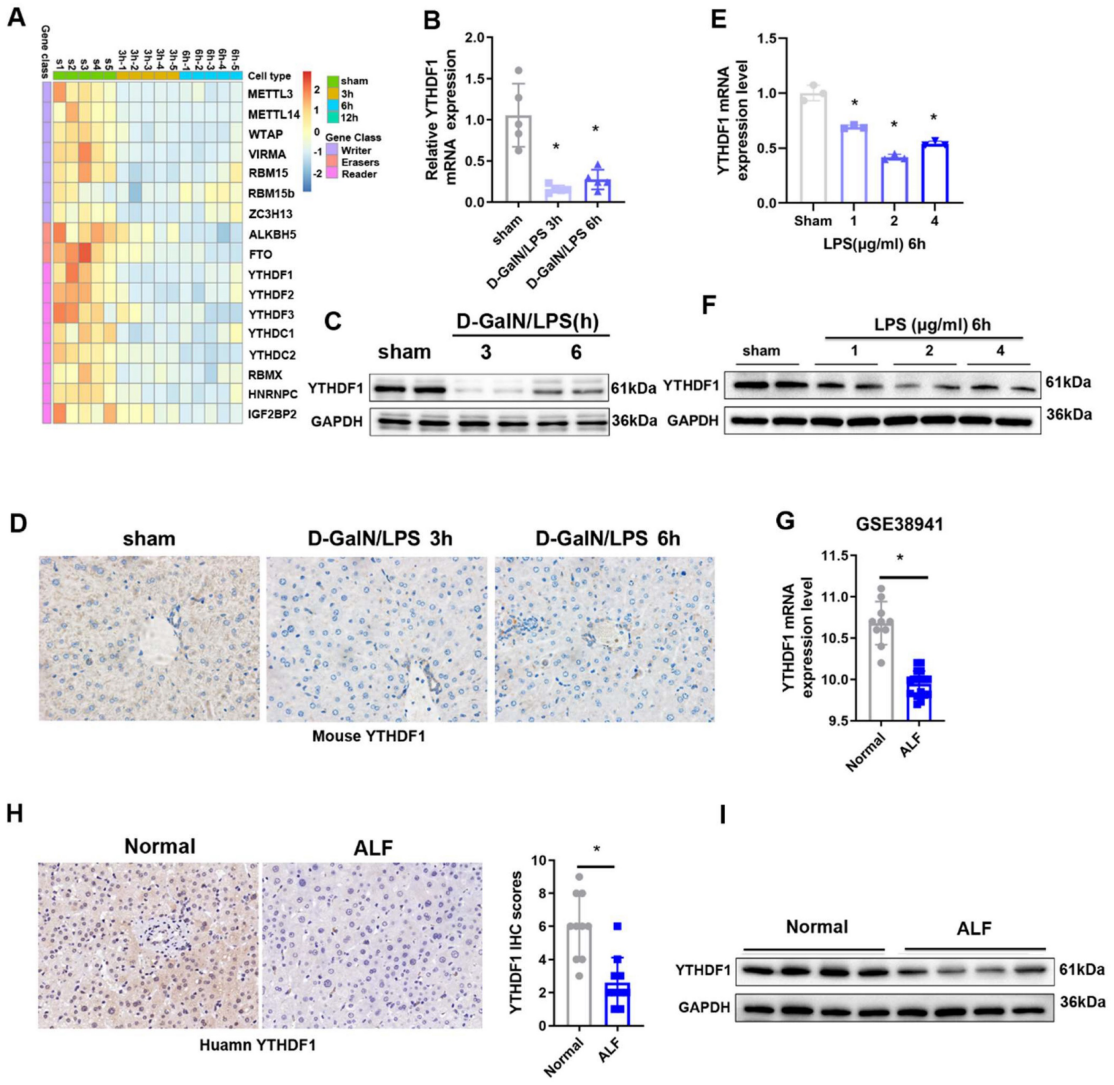
** Decreased expression of YTHDF1 in ALF patients. (A)** Heatmaps showing the mRNA expression of m^6^A-associated genes in the livers of mice subjected to sham treatment or D-GalN/LPS treatment, as determined by qPCR (n=5 per time point). **(B)** Relative YTHDF1 mRNA expression levels (normalized to GAPDH expression) in the livers of mice at 3 and 6h after D-GalN/LPS injection. n = 5 mice in each group.** (C)** YTHDF1 protein expression levels in the livers of mice subjected to D-GalN/LPS for the indicated periods. n = 2 mice in each group.** (D)** Representative IHC staining images showing YTHDF1 expression profiles in the livers of mice subjected to D-GalN/LPS for the indicated periods (400×). n = 4 mice per group; 24 images per mouse.** (E)** RT-qPCR analysis of the gene expression of YTHDF1 relative to GAPDH in primary mouse hepatocytes after LPS stimulation (n = 3 mice per group). **(F)** YTHDF1 protein expression levels in primary mouse hepatocytes after LPS stimulation. n = 2 mice in each group. **(G)** Relative YTHDF1 mRNA expression levels (normalized to GAPDH expression) in the livers of ALF patients according to the GEO database (GSE38941). **(H)** Representative IHC images of YTHDF1 in the explanted livers of 10 patients with ALF and 10 normal livers from patients with hepatic hemangioma (400×).** (I)** Western blot analysis of YTHDF1 protein expression in the explanted livers of patients with ALF (n=4) and normal livers from patients with hepatic hemangioma (n=4). For statistical analysis, two-tailed Student's t test was used. ^*^P < 0.05. In all statistical plots, the data are shown as the mean ± S.D.

To explore the potential role of m^6^A modification in ALF, we first examined the levels of m^6^A in the total RNAs of liver tissues during the ALF process, the data obtained indicated that m^6^A levels in the ALF livers were increased compared to the control group (Supplementary Figure 1A, B). To unbiasedly investigate the participation of m^6^A modification in ALF, A heatmap of the expression data for mRNAs related to m^6^A regulators showed that the expression of most of the m^6^A-related genes was downregulated 3 h and 6 h after D-GalN/LPS insult (Figure 1A). Among these m^6^A related genes, YTHDF1 was the most significantly downregulated m^6^A-related gene, YTHDF1 was decreased by 0.841-fold in the G-DalN/LPS 3h group and 0.725-fold in the and G-DalN/LPS 6h group when compared to sham group (Figure 1B). The change of mRNA level was further confirmed at the protein level by Western blotting and IHC staining (Figure 1C, D). *In vitro*, the mRNA and protein expression of YTHDF1 was also markedly decreased in cultured primary hepatocytes after LPS stimulation (Figure 1E, F).

The above results were recapitulated in the human individuals with fulminant liver injury. YTHDF1 showed the significantly downregulation at the mRNA level in the GEO database (GSE38941) which comprises mRNA data from ALF and normal patients (Figure 1G). Further immunohistochemically (IHC) staining and Western blotting also confirmed that YTHDF1 protein expression was markedly decreased in the group of patients who underwent liver transplantation for hepatitis B virus (HBV)-associated ALF versus the control group (Figure 1H, I). These results indicate that YTHDF1 might be involved in the development of ALF.

### YTHDF1 was specifically decreased in hepatocytes during ALF

We further identified the expression pattern of YTHDF1 in the liver. Differential expression of YTHDF1 (purple/blue color) in the t-SNE projection of mouse liver cells from the single-cell RNA-seq data showed that YTHDF1 was mainly expressed in hepatocytes (Supplementary Figure 2) 26. To further elucidate the precise cell type in which YTHDF1 is expressed during ALF, dual immunofluorescence staining was performed using specific markers of hepatocytes (HNF4α, nuclear), endothelial cells (CD31, cytoplasmic), biliary epithelial cells (CK19, cytoplasmic) and Kupffer cells (KC) plus infiltrating macrophages (F4/80, cytoplasmic). In sham mice, co-staining for HNF4α showed that YTHDF1 was expressed mainly in hepatocytes rather than in the nonparenchymal cells (NPCs). Following D-GalN/LPS injection, YTHDF1 expression was significantly decreased in hepatocytes, whereas the expression of YTHDF1 in NPCs kept stable (Supplementary Figure 3). Considering the specific down-regulation of YTHDF1 in hepatocytes, our subsequent studies mainly focused on this cell type.

### YTHDF1 knockout promoted D-GalN/LPS-induced ALF in murine models

**Figure 2:**
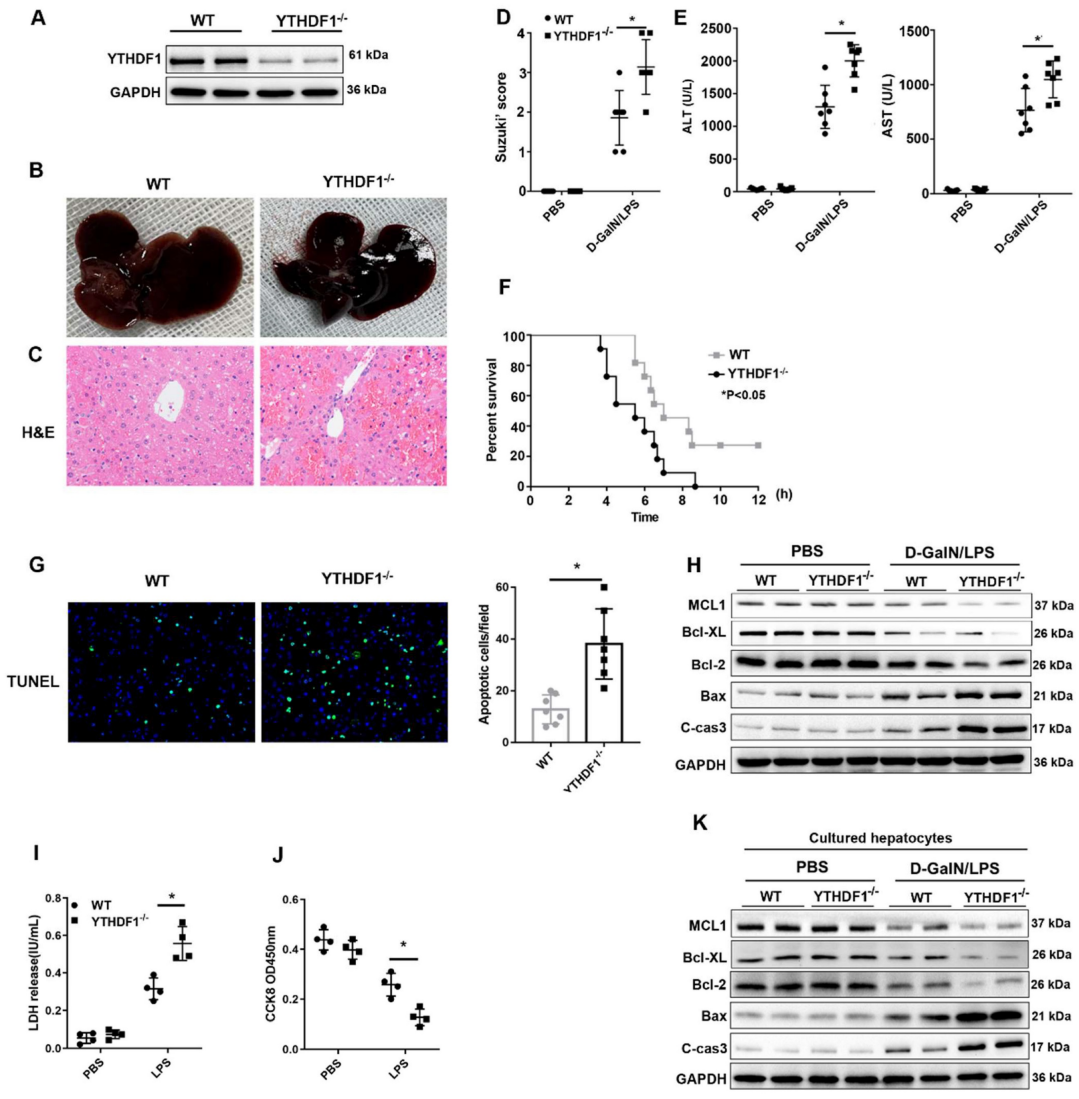
** YTHDF1 knockout aggravated D-GalN/LPS-induced ALF in murine models. (A)** YTHDF1 protein expression in the livers of WT and YTHDF1^-/-^ mice.** (B, C)** Representative images of gross liver morphology and H&E staining of liver sections from WT and YTHDF1^-/-^ mice subjected to sham or D-GalN/LPS treatment (400×; n=7 per group). **(D)** Suzuki scores of the liver sections shown in A (n=7 per group).** (E)** Serum ALT/AST levels of the mice described in B (n=7 per group). **(F)** Survival curves showing the percentages of mouse survival at the indicated times are plotted (n = 11).** (G)** Representative images of TUNEL staining in the liver lobes of WT and YTHDF1^-/-^ mice 6 h after D-GalN/LPS treatment (400×; n=4 per group).** (H)** The protein levels of cell apoptosis-related genes in the livers of mice from the indicated groups after D-GalN/LPS insult (n=2 per group). **(I)** Analysis of the LDH release** (J)** and viability (CCK-8 assay) of hepatocytes isolated from YTHDF1^-/-^ and control mice following treatment with 2 ug/ml LPS for 6 h (n=4 per group). **(K)** Western blotting measuring the protein levels of Bcl-2, Bcl-XL, MCL1, Bax and cleaved caspase-3 in YTHDF1^-/-^ and control hepatocytes after LPS insult (n=2 per group). The data are shown as the mean ± S.D. ^*^P < 0.05.

YTHDF1^-/-^ mice were constructed to evaluate the* in vivo* role of YTHDF1 in ALF. YTHDF1 deficiency in the livers of YTHDF1^-/-^ mice was verified by Western blotting (Figure 2A). D-GalN/LPS were administered to induce ALF model in mice. Six hours after the insult, the livers of YTHDF1^-/-^ mice were more hemorrhagic and swollen than those of WT mouse livers according to gross morphology (Figure 2B). Hematoxylin and eosin (H&E) staining revealed more severe liver injury in YTHDF1^-/-^ mice than in WT mice, as evidenced by increased Suzuki scores (Figure 2C, D). Serum alanine aminotransferase (ALT) and aspartate aminotransferase (AST) levels were significantly higher in YTHDF1^-/-^ mice than in WT mice, indicating that liver function was more severely impaired in YTHDF1^-/-^ mice (Figure 2E). Severe liver injury in YTHDF1^-/-^ mice was associated with a short survival time, and the survival rate of YTHDF1^-/-^ mice was significantly lower than that of WT mice 12 h after D-GalN/LPS administration (0% vs. 27.27%; log-rank (Mantel-Cox) test, *P*=0.025, Figure 2F). Terminal deoxynucleotidyl transferase-mediated deoxyuridine triphosphate nick-end labeling (TUNEL) staining showed increased hepatic apoptosis in YTHDF1^-/-^ mice compared to WT mice (Figure 2G). Consistently, Western blot analysis showed that YTHDF1 knockout caused a marked decline in the expression of the anti-apoptotic protein Bcl-2 Bcl-XL, MCL1 and an increase in the expression of the pro-apoptotic proteins cleaved caspase-3 and Bax (Figure 2H). As such, the* in vivo* study again demonstrated that knockout of the YTHDF1 increased the susceptibility of the mice to D-GalN/LPS-induced ALF.

To further assess the direct protective effect of YTHDF1 against cell death in hepatocytes, primary hepatocytes were isolated from YTHDF1^-/-^ and WT mice and stimulated with LPS (2ug/ml) for 6 h. CCK-8 and LDH assays were used to evaluate cell viability and death. Consequently, YTHDF1 deficiency promoted cell death and suppressed cell proliferation (Figure 2I, J). Moreover, the expression of pro-cell death proteins (Bax and cleaved caspase-3) was increased, while the expression of an anti-apoptosis protein (Bcl-2, Bcl-XL, MCL1) was decreased in YTHDF1-deficient primary hepatocytes compared to control hepatocytes (Figure 2K).

Besides hepatocytes, macrophages play an important role in the inflammatory response in acute liver failure. In the present study, we evaluate the effect of YTHDF1 on macrophages. The data obtained showed that YTHDF1 expression levels were upregulated in RAW264.7 cells after LPS stimulation (Supplementary Figure 4A, B). By using the si-RNA to knock down YTHDF1, the TNF-α and IL-6 mRNA levels were not changed in siYTHDF1-treated RAW 264.7 cells 6 h after LPS stimulation, compared to the NC-treated group (Supplementary Figure 4C-4F), suggesting that YTHDF1 in macrophage may not be involved in LPS stimulation. Collectively, these results indicated that YTHDF1 in hepatocytes exerted a direct effect on cell death during ALF.

### YTHDF1 deficiency compromises mitochondrial homeostasis during D-GalN/LPS-induced ALF

**Figure 3:**
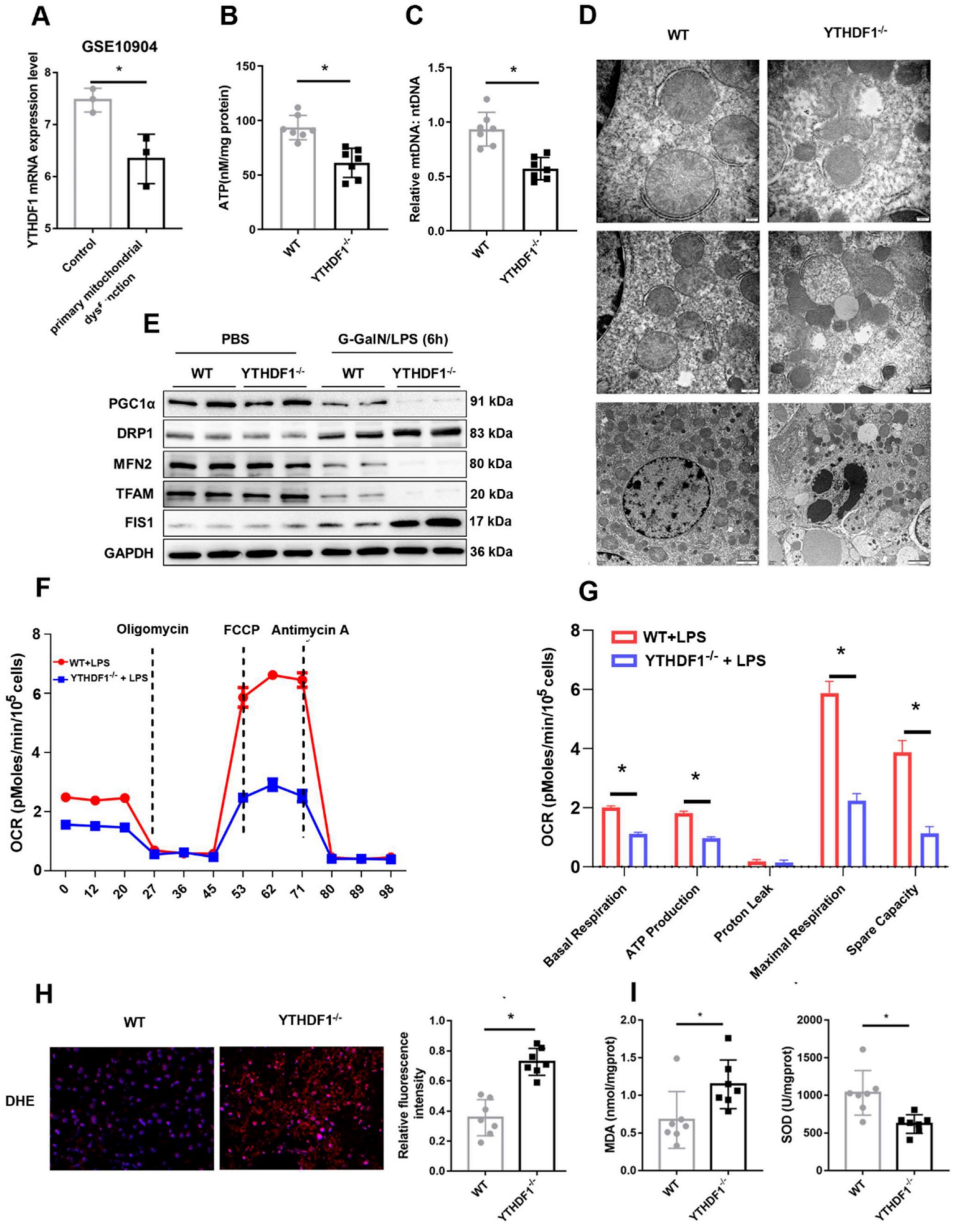
**YTHDF1 deficiency compromises mitochondrial homeostasis in hepatocytes during ALF. (A)** mRNA expression of YTHDF1 in hepatocytes from mice with primary mitochondrial dysfunction according to the GEO database (GSE10904).** (B)** Representative adenosine triphosphate (ATP) level in the liver after ALF. n=7.** (C)** Relative mitochondrial DNA (mtDNA) copy number (mtDNA-to-nDNA). **(D)** Electron microscopy images showing the ultrastructural alterations in the livers of WT and YTHDF1^-/-^ mice 6 h after D-GalN/LPS (n=4 per group).** (E)** Protein expression of PGC-1α, Tfam, Fis-1, Mfn-2 and Drp-1 in the livers of mice from the indicated groups (n=2 per group).** (F-G)** Representative (**F**) and statistical (**G**) results of respiration in WT and YTHDF1^-/-^ mouse primary hepatocytes after LPS stimulation; n = 3.** (H)** Representative images of DHE fluorescence staining in the livers from WT and YTHDF1^-/-^ mice subjected to D-GalN/LPS treatment (400×). **(I)** MDA and SOD levels (n=7 per group).

Mitochondrial dysfunction plays an essential role in the development of ALF^5^, ^27^, ^28^. Downregulation of YTHDF1 expression was observed in a GEO dataset (GSE10904) of a murine model of primary mitochondrial dysfunction (hepatocytes from liver-specific Pdss2 knockout mice) (Figure 3A), suggesting a protective effect of YTHDF1 on mitochondrial function. Next, ATP content analysis also suggested that YTHDF1 deficiency caused a more dramatic decrease in the levels of ATP production (Figure 3B). Consistently, mtDNA copy number was analyzed to examine mitochondrial content. The results showed that the YTHDF1^-/-^ mice had a greater reduction in mtDNA copy number compared with the control group (Figure 3C). Furthermore, mitochondrial morphology was evaluated by transmission electron microscopy. As shown in Figure 3D, mitochondria in KO mice were more severely damaged, showing fragmentation, swelling, and loss of cristae. PGC-1α and Tfam regulate mitochondrial biogenesis, Mfn-2 regulates mitochondrial fusion, and Drp-1 and Fis-1 are important regulators of mitochondrial fission. Western blot analysis and IHC staining showed that YTHDF1^-/-^ mice displayed lower expression levels of PGC-1α, Tfam, and Mfn-2 and higher expression levels of Drp-1 and Fis-1 than WT mice with ALF (Figure 3E and Supplementary Figure 5A-E), confirming the decreased mitochondrial biogenesis by YTHDF1 disruption during ALF. The Seahorse assay indicated a decrease in the oxygen consumption rate (OCR) in YTHDF1^-/-^ mice derived primary hepatocytes compared with control cells (Figure 3F). Moreover, basal respiration, ATP production, maximal respiration and spare capacity were all significantly decreased in YTHDF1^-/-^ mice derived primary hepatocytes compared with control cells (Figure 3G).

In addition, ROS production in the liver, which assessed using dihydroethidine hydrochloride (DHE), was significantly increased in YTHDF1^-/-^ mice (Figure 3H). YTHDF1 knockout mice also exhibited an elevation of malondialdehyde (MDA) activity and a decline of superoxide dismutase (SOD) activity (Figure 3I). Collectively, the results suggested that loss of YTHDF1 in hepatocytes disrupted mitochondrial function, promoted oxidative stress and therefore promoted apoptosis.

### YTHDF1 overexpression mitigated D-GalN/LPS-induced ALF

We then investigated whether YTHDF1 overexpression could in turn alleviate D-GalN/LPS-induced liver injury. We injected C57BL/6 mice with an adeno-associated virus vector expressing YTHDF1 (AAV8- YTHDF1) or empty virus (EV) via the tail vein. Western blotting verified the overexpression of YTHDF1 in the livers of AAV8-YTHDF1 mice (Supplementary Figure 6A). A D-GalN/LPS-induced ALF model was established 4 weeks following AAV8-YTHDF1 injection. As expected, YTHDF1 overexpression in hepatocytes alleviated liver damage (Supplementary Figure 6B-E), hepatocyte apoptosis (Supplementary Figure 6F, G), oxidative stress (Supplementary Figure 6H, I) and mitochondrial structure (Supplementary Figure 6J) induced by D-GalN/LPS. Meanwhile, detection of m^6^A levels shown that administration of D-GalN/LPS induced m^6^A level and alter of YTHDF1 doesn't change m^6^A level (Supplementary Figure 1C-F). Taken together, these findings suggested that YTHDF1 overexpression alleviated D-GalN/LPS-induced ALF.

### Transcriptome-wide m^6^A-seq, RNA-seq and RIP-seq assays identify potential targets of YTHDF1 in HCC

**Figure 4:**
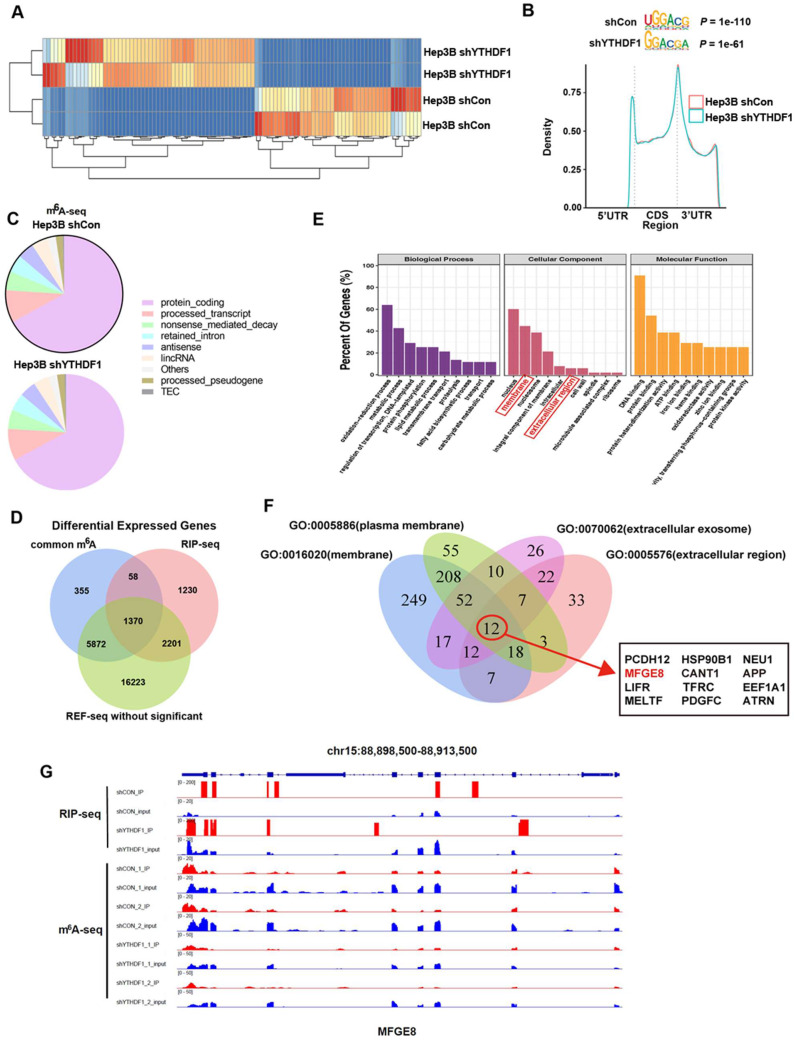
** Identification of YTHDF1 targets in liver cells. (A)** Heatmap of differentially expressed genes (DEGs) identified by RNA-seq.** (B)** Enriched consensus motifs were detected within m6A peaks. Statistical analyses were performed using a one-tailed binomial test. Metagene distribution of the m6A peaks of YTHDF1-knockdown and control Hep3B cells throughout the transcriptome. **(C)** Proportion of m^6^A peak distribution in the 5'UTR, start codon, CDS, stop codon and 3'UTR among the entire set of mRNA transcripts. **(D)** Venn diagram illustrating the overlapping genes identified by m^6^A-seq, RIP-seq, and RNA-seq. Common m6A represents genes could be labeled with m6A. **(E)** GO enrichment analysis of genes described in (D). **(F)** Venn diagram narrowing the overlapping genes involved in secretion function. **(G)** IGV tracks displaying m^6^A peaks and YTHDF1 binding enrichment in MFG-E8 from m^6^A-seq and YTHDF1 RIP-seq in HCC cells.

We next explored the mechanisms underlying the protective role of YTHDF1 against ALF. YTHDF1-knockdown and control Hep3B cells were firstly subjected to RNA-seq analysis. YTHDF1 depletion resulted in expression alteration of 320 genes globally, including 124 upregulated genes and 196 downregulated genes (Figure 4A). As an m^6^A reader, YTHDF1 works *by* binding m^6^A methylated transcripts.

Thus, we performed antibody-based m^6^A profiling (m^6^A-seq) and RNA immunoprecipitation and sequencing (RIP-seq) in YTHDF1-knockdown and control Hep3B cells. By applying the HOMER motif discovery tool, we found that the “GGAC” consensus sequence was the primary motif enriched in the m^6^A peaks (Figure 4B). In line with prior studies, we found that YTHDF1 knockdown did not change the m^6^A peak density significantly. Peaks were mainly located in protein-coding transcripts and were abundant around stop codons (Figure 4B, C). Interestingly, overlapping the genes from m^6^A-seq, and RIP-seq revealed that 1422 genes with m^6^A modification were also bound by YTHDF1, among which 1370 genes were not altered upon YTHDF1 knockdown, as shown by RNA-seq (Figure 4D). In addition, Gene Ontology (GO) enrichment analysis revealed that these 1370 genes were related to these top GO terms, including nucleus, membrane, nucleosome, integral component of membrane, intracellular, extracellular region, cell wall, spindle, microtubule associated complex and ribosome (Figure 4E).

More and more researches revealed the vital role of hepatokines in regulating liver diseases and potential therapeutic effects 29-31. Thus, we decided to find which of these transcripts could be secreted. A Venn plot showed that the overlapping genes with GO terms from GO: 0005886 (plasma membrane), GO: 0016020 (membrane), GO: 0070062 (extracellular exosome) and GO: 0005576 (extracellular region) narrowed down to 12 candidate genes involved in secretion function (Figure 4F, Supplementary Table 3). Of these candidate genes, MFG-E8 attracted our attention. MFG-E8 is an endogenous protective mediator in acute injury models, including acute lung injury, acute pancreatitis, sepsis and ischemia-reperfusion injury^32^-^34^. More importantly, recombinant MFG-E8 protein presents a potential therapeutic option for a variety of acute injury models. We evaluated the alterations in MFG-E8 expression in liver samples during ALF to investigate the potential role of MFG-E8. Analysis of the ALF GEO database (GSE38941) revealed no significant difference in MFG-E8 expression at the mRNA level between normal subjects and patients with ALF (Supplementary Figure 7A). However, MFG-E8 protein levels were markedly decreased in both clinical ALF samples (Supplementary Figure 7B, C) and a murine ALF model (Supplementary Figure 7D-F), as indicated by IHC staining and Western blotting. These results suggested that MFG-E8 may play a potential epigenetic role in the pathogenesis of ALF.

We further analyzed the distribution of m^6^A peaks and RIP peaks of YTHDF1-binding mRNAs and found that the m^6^A peaks were located coincident with the YTHDF1-binding enrichment region (m^6^A-seq+RIP-seq) in MFG-E8, as shown by Integrative Genomics Viewer (IGV) software. These data indicated that MFG-E8 is an important and direct target of YTHDF1 (Figure 4G).

### YTHDF1 promoted the translation efficiency of MFG-E8

**Figure 5:**
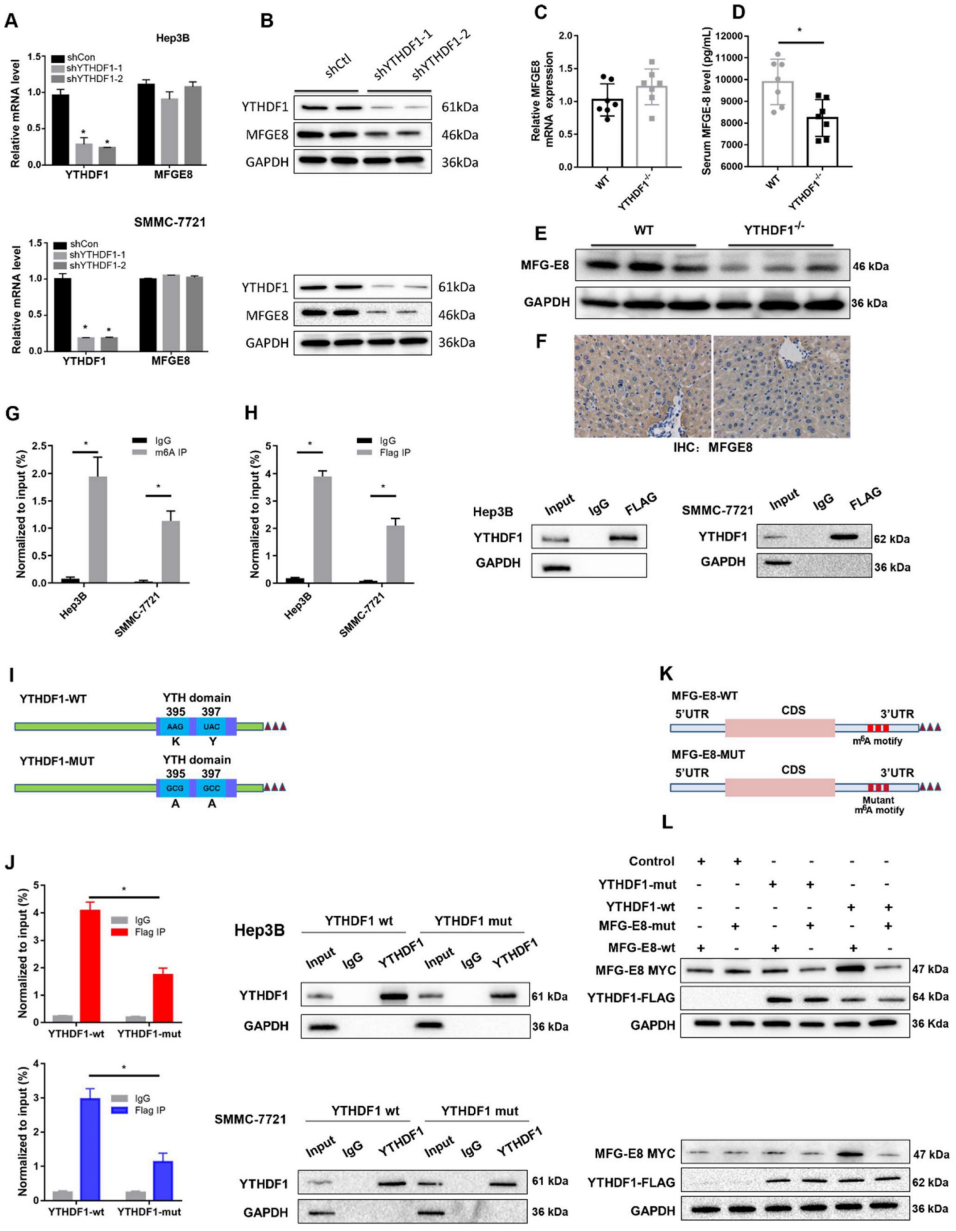
** YTHDF1 regulated MFG-E8 expression in HCC cells in an m^6^A methyltransferase-dependent manner. (A)** The relative mRNA expression of MFG-E8 in Hep3B and SMMC-7721 cells upon YTHDF1 knockdown. **(B)** Protein level of MFG-E8 in Hep3B and SMMC-7721 cells upon YTHDF1 knockdown. **(C)** Relative mRNA expression levels of MFG-E8 in the livers of WT mice and YTHDF1^-/-^ mice 6 h after D-GalN/LPS insult. Gene expression was normalized to the expression of GAPDH. n = 7 mice per group.** (D)** Serum MFG-E8 levels in WT mice and YTHDF1^-/-^ mice 6 h after D-GalN/LPS insult. n = 7 mice per group. **(E** and** F)** Representative IHC staining and Western blot analysis of MFG-E8 protein in the livers shown in D (400×, n=3 per group). **(G)** Gene-specific m^6^A qPCR validation of m^6^A levels in Hep3B and SMMC-7721 cells (n=3). **(H)** RIP-qPCR confirmed the interaction between YTHDF1 and MFG-E8 mRNA; the data are expressed relative to the input levels (n=3). Western blotting of precipitated proteins in the endogenous YTHDF1 RIP assay. **(I)** Schematic representation of the YTHDF1-wt and YTHDF1-mut constructs **(J)** RIP-derived RNA and protein levels in Hep3B and SMMC-7721 cells were measured by RT-qPCR and Western blotting, respectively. GAPDH was used as the negative control for Western blot analysis.** (K)** Schematic representation of the MYC-tagged wild-type (MFG-E8-wt) and mutant (MFG-E8-mut) MFG-E8 constructs.** (L)** Western blot analysis confirmed that MYC-tagged MFG-E8 was expressed in Hep3B and SMMC-7721 cells cotransfected with empty vector, wild-type or mutant Flag-tagged YTHDF1 and MYC-tagged wild-type or mutant MFG-E8. The data are shown as the mean ± S.D. *P < 0.05.

The data obtained from qPCR and Western blotting indicated that loss of YTHDF1 suppressed MFG-E8 expression at protein level without affecting its mRNA level in both Hep3B and SMMC-7721 cells (Figure 5A-B). Consistently, MFG-E8 protein was significantly reduced in the liver of YTHDF1 deficient mice compared to that in the littermates while its mRNA level remained similar in ALF model mice (Figure 5C-F). These results indicated that YTHDF1 promotes MFG-E8 expression in the manner of post-transcriptional modification. We further treated cells with CHX to evaluate the effect of YTHDF1 on the translation efficiency of MFG-E8. The western blot analysis presented that MFG-8 protein levels were significantly higher in YTHDF1 overexpressing cells as compared to controls, which was abrogated in the CHX treatment group (Supplementary Figure 8A). The result suggested that the increased MFG-E8 protein level is achieved by YTHDF1-mediated enhanced translational efficiency.

To further confirm the regulatory effect of YTHDF1 on MFG-E8, IHC staining for YTHDF1 and MFG-E8 was performed using 99 HCC tissue samples. As shown in Supplementary Figure 8B, C, 26 (26.27%) HCC samples with positive staining of YTHDF1 showed intense MFG-E8 staining, while 34 (34.34%) with faint YTHDF1 staining exhibited lower levels of MFG-E8 expression (*P*=0.015). These findings indicated that YTHDF1 activation positively affects MFG-E8 expression in HCC.

### YTHDF1-mediated translation of MFGE-8 mRNA required m^6^A modification of the transcript

Furthermore, a gene-specific m^6^A assay was used to determine the m^6^A modification status of MFG-E8 mRNA, and the results showed that MFG-E8 mRNA was significantly enriched in m^6^A modification (Figure 5G). Moreover, RIP-qPCR confirmed that YTHDF1 could directly interact with MFG-E8 mRNA (Figure 5H). Previous studies have reported that YTHDF1 binds to m^6^A sites through m^6^A-binding pockets in the YTH domain and that mutation of the K395 and Y397 residues can prevent the binding between YTHDF1 and mRNAs.

To test whether YTHDF1-mediated regulation of MFG-E8 expression was m^6^A dependent, we constructed mutant YTHDF1 with K395A and Y397A mutations in the YTH domain (YTHDF1-mut) and then transfected liver cells with the YTHDF1-mut or YTHDF1 wild-type (YTHDF1-wt) construct (Figure 5I). Subsequent RIP-qPCR demonstrated that MFG-E8 mRNA was successfully immunoprecipitated from cells transfected with YTHDF1-wt but not YTHDF1-mut (Figure 5J), suggesting that the m^6^A-binding pocket is crucial for the binding of YTHDF1 to MFGE-8 mRNA. Meanwhile, we constructed a MYC-tagged MFG-E8 expression vector (MFG-E8-wt) and a vector expressing MFG-E8 with a mutation in m^6^A sites (MFG-E8-mut) (Figure 5K). Western blot analysis revealed that YTHDF1-mut had lost its effect in promoting MFG-E8 mRNA translation, and mutations within the m^6^A peak had no response to wild-type YTHDF1 overexpression (Figure 5L). Collectively, these data suggested that YTHDF1 induced MFG-E8 protein expression in an m^6^A-dependent manner.

### Genetic deletion of MFG-E8 aggravated hepatic injury in mice with ALF

**Figure 6:**
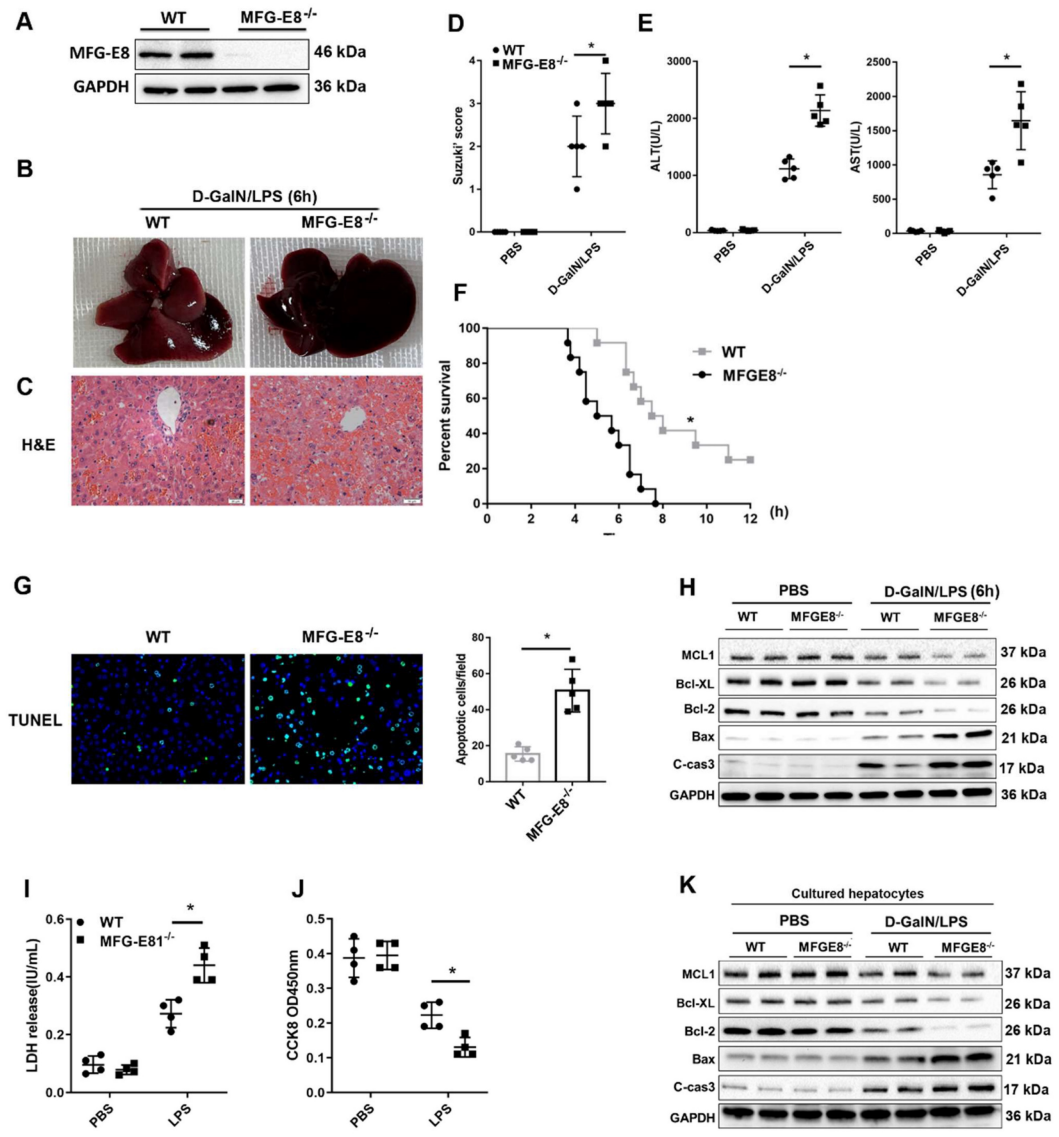
** Genetic deletion of MFG-E8 aggravated hepatic injury in ALF mice. (A)** MFG-E8 protein expression in the livers of WT and MFG-E8^-/-^ mice, n=4 per group. **(B-C)** Representative images of gross liver morphology **(B)** and H&E staining **(C)** from WT and MFG-E8^-/-^ mice subjected to sham or D-GalN/LPS treatment (400×, n=4 per group). **(D, E)** Suzuki scores of liver sections **(D)**, Serum ALT/AST activity** (E)** in the WT and MFG-E8 knockout groups 6 h after D-GalN/LPS (n=5 per group).** (F)** Lethal effect of D-GalN/LPS in mice (n=12 per group). The percentages of surviving mice at the indicated times were plotted (log-rank with Mantel-Cox test).** (G)** Representative images of TUNEL staining in the liver lobes of WT and MFG-E8^-/-^ mice 6 h after D-GalN/LPS treatment (400×; n=4 per group).** (H)** The protein levels of cell death-related genes in the livers of the indicated mouse groups after D-GalN/LPS insult (n=2 per group). Analysis of the LDH release**(I)** and viability (CCK-8 assay)** (J)** of hepatocytes isolated from MFG-E8 knockout and control mice following treatment with LPS for 6 h. The bar graph shows data from three independent experiments. **(K)** Western blot analysis of the protein levels of Bcl-2, Bcl-XL, MCL1, Bax and cleaved caspase-3 in MFG-E8 knockout and control hepatocytes after LPS insult. n=2 per group. The data are shown as mean ± S.D. *P < 0.05.

To explore the* in vivo* function of MFG-E8 in ALF, we generated conventional MFG-E8 knockout (MFG-E8^-/-^) mice (Figure 6A). As expected, the livers of knockout mice became engorged with blood after D-GalN/LPS administration (Figure 6B). Histological analysis of the livers also showed increased Suzuki score in MFG-E8^-/-^ mice compared to WT mice (Figure 6C and D). Serum biochemistry tests displayed a significant increase in serum ALT and AST levels in the livers of MFG-E8^-/-^ mice compared with those of WT mice (Figure 6E). Kaplan-Meier survival analysis also indicated that MFG-E8^-/-^ mice exhibited worse overall survival (log-rank (Mantel-Cox) test, *P*=0.0008) than control mice (Figure 6F). There were markedly more TUNEL-positive cells in the livers of knockout mice than in the livers of WT mice of after treated with D-GalN/LPS (Figure 6G). Moreover, Western blot analysis of the expression of apoptosis-associated proteins showed the same results (Figure 6H). Thus, these results confirmed the hypothesis that inhibition of MFG-E8 aggravates the development of ALF in mice. In line with the* in vivo* results, *in vitro* experiments revealed that stimulation of primary hepatocytes with LPS induced LDH release and reduced cell proliferation and that MFG-E8 inhibition worsened these phenotypes (Figure 6I, J). Additionally, LPS-induced cell death was drastically aggravated in MFG-E8-deficient primary hepatocytes, as evidenced by obvious changes in the expression of apoptotic regulators (Figure 6K).

**Figure 7:**
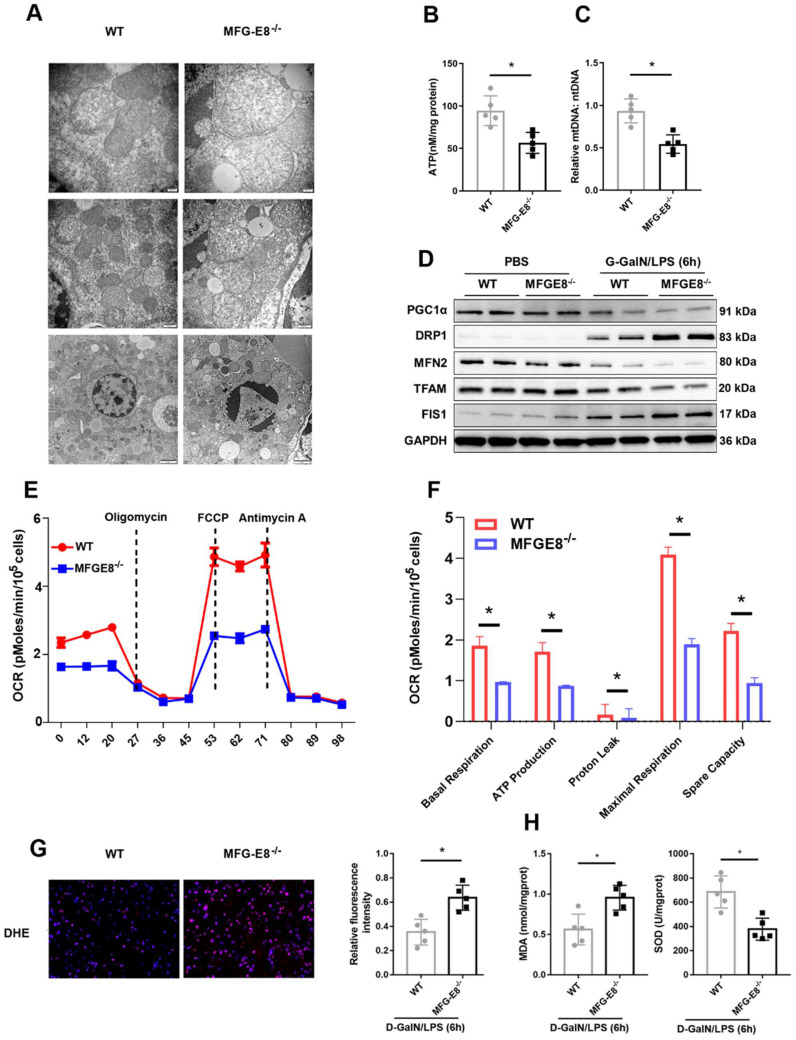
** MFG-E8 deficiency impaired mitochondrial function and increased oxidative stress in experimental ALF. (A)** Ultrastructural images showing mitochondrial morphology. **(B)** ATP levels in the livers.** (C)** Relative mtDNA-to-nDNA levels in the livers. **(D)** Western blot analysis of the expression of PGC-1α, Tfam, Fis-1, Mfn-2 and Drp-1 in the livers of the indicated mouse groups, n=2 per group. **(E-F)** Representative (**E**) and statistical (**F**) results of respiration in WT and MFG-E8^-/-^ mouse primary hepatocytes after LPS stimulation; n = 3. **(G)** Representative images of DHE fluorescence staining in the livers of WT and MFG-E8^-/-^ mice (400×, n=4 per group). **(H)** MDA and SOD levels (n=5 per group).** (J)** Ultrastructural images showing mitochondrial morphology.

We further determined whether MFG-E8 deletion can exacerbate mitochondrial dysfunction. We observed more serious mitochondrial damage in MFG-E8^-/-^ mice through TEM (Figure 7A). ATP content and mtDNA copy number were decreased markedly in MFG-E8^-/-^ mice in compared with the control group (Figure 7B, C). Western blot analysis showed that MFG-E8^-/-^ mice displayed lower expression levels of PGC-1α, Tfam, and Mfn-2 and higher expression levels of Drp-1 and Fis-1 than WT mice with ALF (Figure 7D). The Seahorse assay showed a decrease in the OCR in MFG-E8-deficient primary hepatocytes compared with control cells (Figure 7E). Basal respiration, ATP production, proton leakage, maximal respiration and spare capacity were all significantly decreased in MFG-E8-deficient primary hepatocytes compared with control cells (Figure 7F). In addition, knockout mice had higher fluorescence intensity of DHE staining, hepatic MDA levels and lower SOD levels than WT mice (Figure 7G, H). Thus, these results indicated that depletion of MFG-E8 accelerated mitochondrial dysfunction and ROS production in hepatocytes.

### MFG-E8 mediated the function of YTHDF1 in ALF

**Figure 8:**
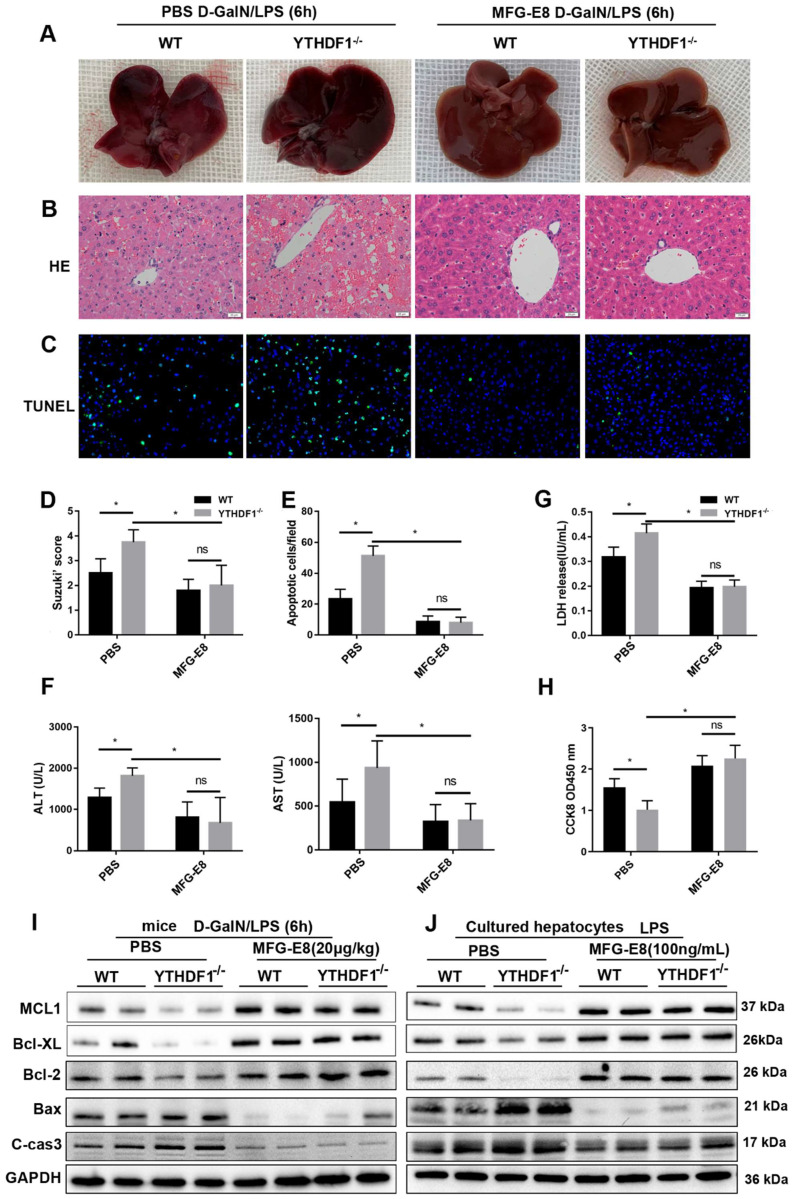
** Recombinant MFG-E8 protein treatment abolished the detrimental effect of YTHDF1 knockdown during ALF.** YTHDF1^-/-^ and WT mice were injected with recombinant MFG-E8 or vehicle (PBS) prior to D-GalN/LPS insult (n=5 per group). **(A-C)** Representative images of gross liver morphology** (A)** and H&E staining **(B)** and TUNEL staining **(C)** of liver sections from WT and YTHDF1^-/-^ mice pretreated with recombinant MFG-E8 or vehicle (PBS) subjected to D-GalN/LPS for 6 h (400×).** (D-F)** Suzuki scores of liver sections **(D)**, quantitative analysis of apoptotic cells **(E)** and serum ALT/AST levels **(F)** in the indicated groups (n=5 per group). **(G and H)** CCK-8 assay and LDH release of primary hepatocytes isolated from YTHDF1^-/-^ and WT mice treated with recombinant MFG-E8 or vehicle (PBS). **(I)** Western blotting showing the protein levels of Bcl-2, Bcl-XL, MCL1, Bax and cleaved caspase-3 in YTHDF1^-/-^ and WT mice subjected to recombinant MFG-E8 or vehicle injection 2 h before D-GalN/LPS insult (n=2 per group). **(J)** Bcl-2, Bcl-XL, MCL1, Bax and cleaved caspase-3 protein expression in isolated hepatocytes of YTHDF1^-/-^ and WT mice treated with recombinant MFG-E8 or vehicle was analyzed by Western blotting (n=2 per group). The data are shown as the mean ± S.D.; ^*^P < 0.05; ns indicates no significance.

Recombinant MFG-E8 protein was injected into YTHDF1^-/-^ and WT mice *via* the tail vein to further verify the necessity of MFG-E8 in the protective effect of YTHDF1 against ALF. Gross liver specimens, histological analysis, serum aminotransferase (ALT and AST) activity and TUNEL staining revealed that treatment with recombinant MFG-E8 protein significantly protected against liver damage in both YTHDF1^-/-^ and WT mice. More importantly, no significant difference in liver damage or cell death was observed between YTHDF1^-/-^ and WT mice treated with recombinant MFG-E8 protein (Figure 8A-F). In addition, recombinant MFG-E8 protein administration abrogated the detrimental effect of YTHDF1 deficiency by rescuing hepatocyte proliferation and suppressing LDH release in primary hepatocytes (Figure 8G, H). Western blot analysis showed that treatment with recombinant MFG-E8 protein mitigated cell death *in vivo* and* in vitro*, as evidenced by increased Bcl-2, Bcl-XL, MCL1 expression and decreased cleaved Caspase 3 and Bax levels (Figure 8I, J). These results demonstrated that restoration of MFG-E8 completely abrogated the effect of YTHDF1 deficiency in ALF and suggested that MFG-E8 mediates YTHDF1 function in ALF.

It has been reported MFG-E8 restores mitochondrial function in acute pancreatitis 32. To explore whether the protective effect of YTHDF1 on mitochondrial function is dependent of MFG-E8, further data of evaluating the mitochondrial function presented that administration of MFG-E8 eliminating the destructive effect resulted from YTHDF1 knockout, as evidenced by the upregulation of ATP level and mtDNA copy number in mice (Supplementary Figure 9A, B) and reversed mitochondrial respiratory capacity in hepatocytes (Supplementary Figure 9C). Western-blot assay of mitochondrial fission and fusion related proteins in mice and hepatocytes consistently indicated that treatment of MFG-E8 suppressed the effect of YTHDF1 deficiency on ALF-derived increases in mitochondrial damage (Supplementary Figure 9D, E).

### YTHDF1 modulated MFG-E8- FAK-STAT3 signaling in ALF

A previous study suggested that MFG-E8 improves mitochondrial function by activating the integrin-mediated FAK-STAT3 signaling pathway in acute pancreatitis 32. Thus, we next explored whether YTHDF1 restores mitochondrial function via MFG-E8-FAK-STAT3 signaling in the present study. In addition, the phosphorylation of FAK and STAT3 was inhibited in shYTHDF1 cells and YTHDF1^-/-^ mice (Supplementary Figure 10). Thus, YTHDF1 protected against ALF, possibly by restoring mitochondrial function via activation of the MFG-E8-FAK-STAT3 signaling pathway.

## Discussion

It has been reported that m^6^A is involved in antitumor immunity 35, tumorigenesis 36, metastasis 11, 37, heat shock response 38, and hematopoietic stem and progenitor cell specification^39^. However, its role in acute liver injury remains unknown. As such, the current study is important, as it demonstrated for first time that m^6^A was critically involved in the development of ALF. In fact, among all the m^6^A-modified genes, YTHDF1 was the most significantly involved in ALF. Through genetic manipulation, we showed that YTHDF1 deficiency exacerbated D-GalN/LPS-induced liver injury, whereas YTHDF1 overexpression protected against liver injury. YTHDF1 was identified as a critical role of in the development of ALF.

Previous studies have highlighted that mitochondrial dysfunction is essential for ALF. Oner et al. demonstrated that mitochondrial dysfunction was an important cause of damage in APAP toxicity^40^. Naroa et al. reported that affected mitochondrial respiration and ATP production are was the leading cause of early posttransplantation organ failure^41^. In addition, the mitochondria are the primary source of reactive oxygen species (ROS) under pathological condition, and ROS is a critical detrimental factor promoting the progression of D-GalN/LPS -induced liver injury. However, the biological function of m^6^A modification in mitochondrial function remained unknown. Herein, we demonstrated that YTHDF1 protected against D-GalN/LPS -induced liver injury by alleviating mitochondrial dysfunction, oxidative stress and cell apoptosis* in vivo* and *in vitro*.

Mechanistically, to dissect the mechanisms of YTHDF1 in ALF at the molecular level, we performed multiomics screening by combining m^6^A-seq and RIP-seq as well as RNA-seq. We found that high enrichment of m^6^A in the 3′UTR of MFG-E8 mRNA promotes the translation of MFG-E8 mRNA by binding of the m^6^A reader YTHDF1, revealing a novel m^6^A-dependent posttranscriptional mechanism of MFG-E8 expression. MFG-E8 is a secreted lipophilic glycoprotein that contains an RGD motif, which is known to interact with integrins^42^. Serum MFG-E8 expression has been shown to be downregulated under acute inflammatory conditions such as sepsis and ischemia-reperfusion injury, and MFG-E8 has been found to play an important role in protecting against sepsis, attenuating inflammatory responses and tissue injury 33, 34. We found decreased protein expression of MFG-E8 without a corresponding decrease in mRNA levels in the context of ALF. Knockdown of MFG-E8 resulted in exacerbation of cellular apoptosis and disruption of mitochondrial function. MFG-E8 knockout mice suffered more severe liver injury and greater mitochondrial damage than WT mice after D-GalN/LPS administration. Therefore, MFG-E8 might act a vital regulator in ALF. In addition, phenotypic examination indicated that administration of recombinant MFG-E8 protein reversed the destructive effect of YTHDF1 deficiency on D-GalN/LPS-induced liver damage. Thus, YTHDF1-mediated regulation of ALF probably depends on the activation of MFG-E8. Administration of recombinant MFG-E8 may be a promising therapeutic strategy for the prevention and treatment of ALF.

STAT3 is an important regulator of mitochondrial function. Phosphorylation of STAT3 can stimulate mitochondrial bioenergetic function, and integrin-FAK-STAT3 signaling promotes mitochondrial function 43. A previous study also showed that MFG-E8 alleviates acute pancreatitis by improving mitochondrial function via activation of the integrin-FAK-STAT3 signaling pathway 32. In the present study, the phosphorylation of FAK and STAT3 was inhibited in shYTHDF1 cells, whereas recombinant MFG-E8 protein treatment increased the levels of phosphorylated FAK and STAT3 to almost the levels observed in the control group. Our data suggest that YTHDF1 protects against D-GalN/LPS-induced mitochondrial dysfunction in the mouse liver through activation of the MFG-E8-FAK-STAT3 signaling pathway.

Considering that L02 cells were contaminated by Hela cells, the high-throughput sequencing used Hep3B cells which have been widely used in other studies 5. In addition, we reproduced the biological function in primary mouse hepatocytes, and the YTHDF1 -MFG-E8 signal path was also verified by mouse tissue specimen.

In conclusion, our results demonstrated that the m^6^A reader YTHDF1 protected against D-GalN/LPS-induced apoptosis in the liver. We also identified MFG-E8 as the direct target of YTHDF1. YTHDF1 regulated MFG-E8 translation in an m^6^A-dependent manner and restored mitochondrial function in ALF. We did not exclude the potential protective functions of other targets of YTHDF1 or other m^6^A regulators in ALF, and further investigation of the complex regulatory pathways mediated by the m^6^A axis is necessary. Our findings shed new light on the development and progressions of ALF associated with m^6^A/YTHDF1 deregulation and suggest that activation of YTHDF1 or administration of MFG-E8 may be a potential therapeutic strategy for ALF patients.

## Materials and Methods

### Animals

The YTHDF1 knockout (YTHDF1^-/-^) mice were generated by CRISPR-cas9-mediated genome editing technology (Cyagen Biosciences). MFG-E8 knockout (MFG-E8^-/-^) mice were generated by knocking out 2-6 exons of MFG-E8 gene using CRISPR/Cas9 gene editing technology (Shanghai Model Organisms, Supplementary Figure 11). The primer sequences used were presented in supplementary table 1. Animal studies were carried out in a specific pathogen-free (SPF) animal facility, and littermates were also cohoused during experiments to reduce variation in the microbiome and environment. All animal care and experimental procedures were approved by the Ethics Committee of Xi'an Jiaotong University, according to the National Health Guidelines for the Care and Use of Laboratory Animals.

### ALF model

The ALF mouse model was established as previously described 44. The mice were intraperitoneally injected with D-GalN (750 mg/kg; Sigma, St. Louis, MO, USA) and LPS (10 μg/kg; Sigma, St. Louis, MO, USA) to induce acute liver failure. For intervention study, the mice were intraperitoneally administered MFG-E8 (20 μg/kg, RD System, Inc. Mnnesota, USA) two hours before the construction of the D-GalN/LPS-induced acute liver failure. The doses of MFG-E8 used were chosen as previously reported 32. The mice were anesthetized with isoflurane inhalation at 6 h after D-GalN/LPS injection before blood samples and liver tissues were harvested.

### Clinical samples and tissue microarray

Paraffin-embedded samples of resected tumors from 99 HCC patients collected at the First Affiliated Hospital of Xi'an Jiaotong University from June 2014 - July 2018 were obtained. Clinical information was described in the prior study 45.

Human liver samples were collected from patients with HBV-associated ALF undergoing liver transplantation and patients with hepatic hemangioma undergoing liver resection. The diagnosis of ALF was based on the occurrence of liver failure and hepatic encephalopathy within 8 weeks of the onset of the first symptoms in individuals without prior liver disease. All procedures performed in studies involving human participants were in accordance with the ethical standards of the Research Ethics Committee of The First Affiliated Hospital of Xi'an Jiaotong University (2017-122) and with the 1964 Helsinki declaration and its later amendments. All written informed consent documents were obtained from patients for samples collection.

### Statistical analysis

Data are expressed as the mean ± SD. Statistical analysis was performed using GraphPad Prism 9 software (GraphPad Software, Inc.) Two-tailed unpaired Student's t test or Pearson chi-squared test was used for comparisons between groups when appropriate. Overall survival was evaluated by the Kaplan-Meier method and log-rank test. ^*^p < 0.05 was considered statistically significant.

More details of the methods are available in the Supplementary Materials.

## Supplementary Material

Supplementary materials and methods, figures and tables.
