# Supplementary material for: The m6A reader YTHDF1 attenuates fulminant hepatitis via MFG-E8 translation in an m6A dependent manner
Source: Int J Biol Sci. 2023 Jul 31;19(12):3987–4003. doi: 10.7150/ijbs.84768 (PMC10411475; doi:10.7150/ijbs.84768)
Supplement: Supplementary file 1 — Supplementary materials and methods, figures and tables. [file ijbsv19p3987s1.pdf]

Supplementary Materials

Supplementary Figures

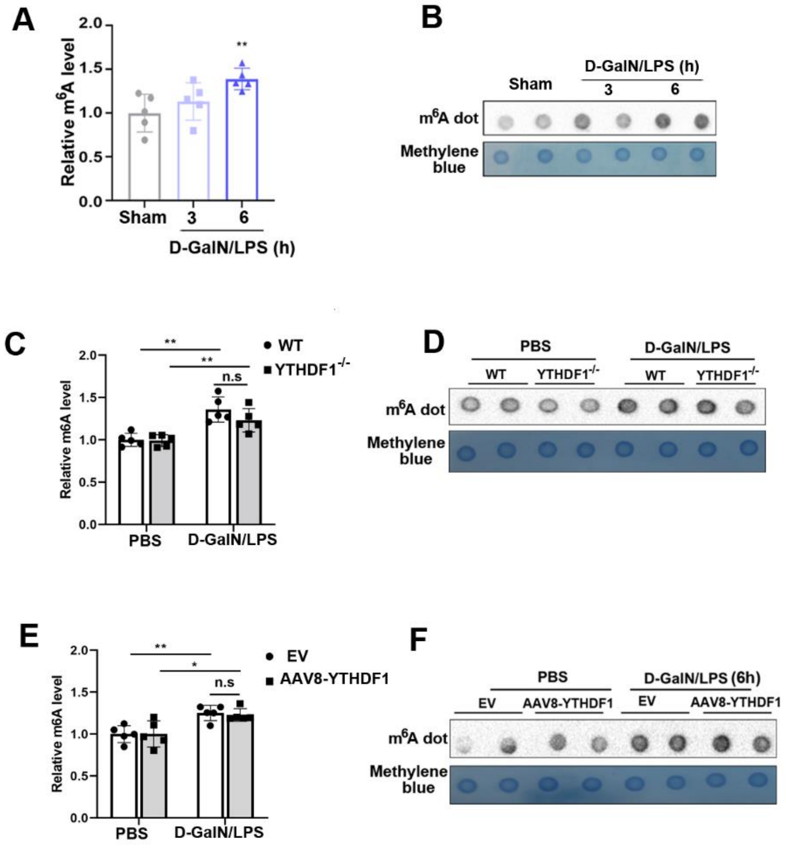

Figure S1. The levels of m^{6}A in ALF process. (A, C, E) The m^{6}A contents of total RNAs in liver tissues in different groups. (B, D, F) RNA dot blot analysis of m^{6}A levels in each group. Methylene blue staining serves as a loading control. Forstatistical analysis, two-tailed Student's t test was used. *P<0.05, **P<0.01. In all statistical plots, the data are shown as the mean ± S.D, ns indicates no significance.

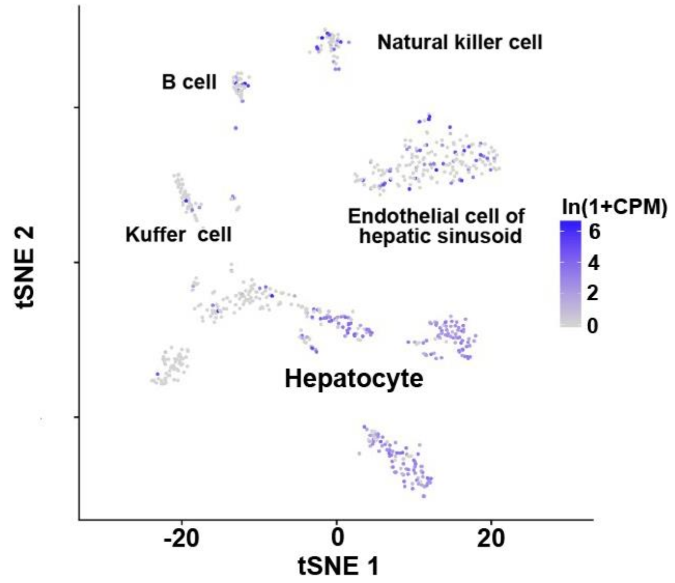

Figure S2. Expression of YTHDF1 in liver cells. (A) Differential expression of YTHDF1 (purple/blue color) in t-SNE projection of mouse liver cells from the single-cell RNAseq data (Tabula Muris Consortium et al., 2018).

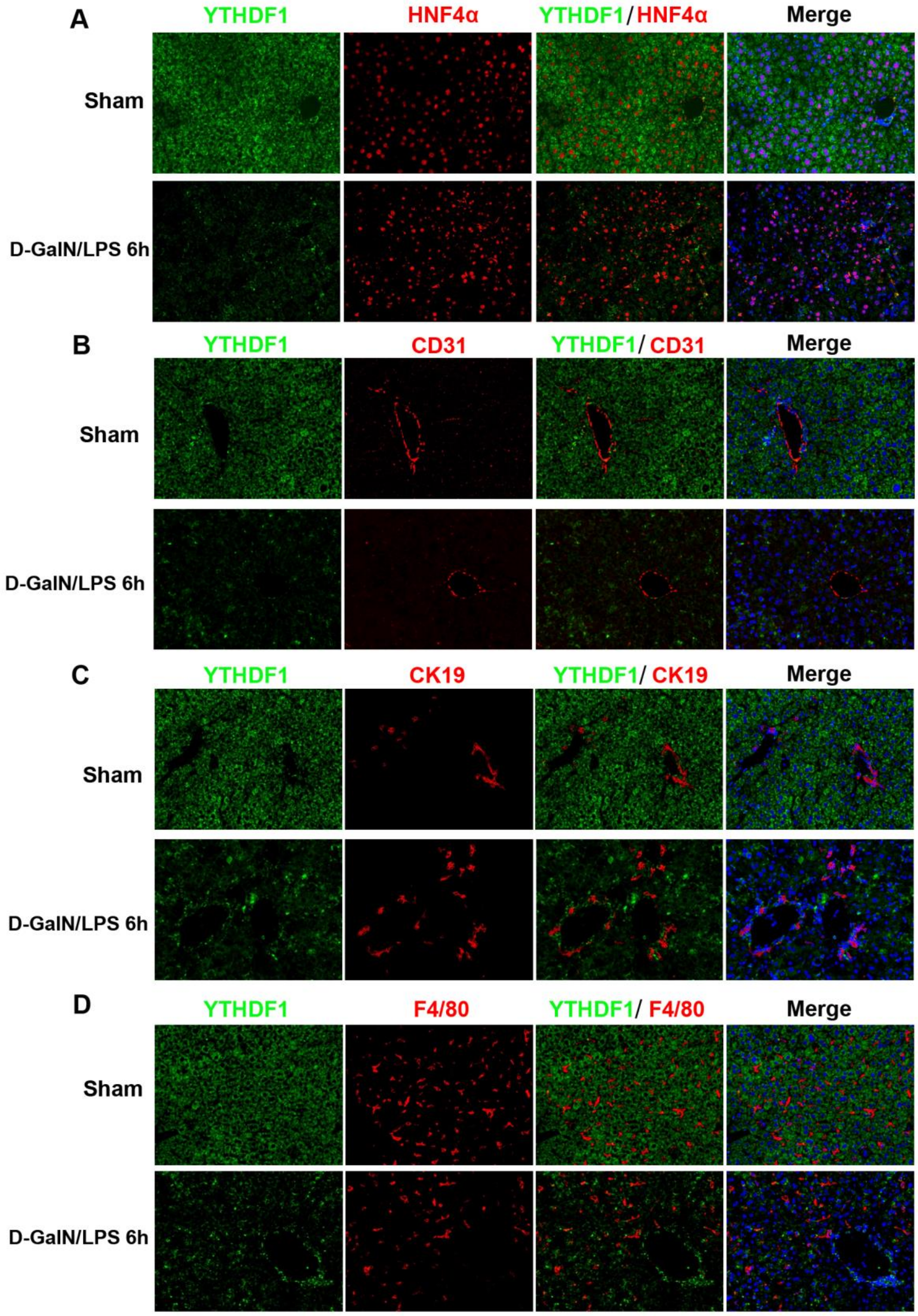

Figure S3. Identification of the cellular source of YTHDF1 in ALF. Double IF staining of liver sections showing colocalization of YTHDF1 (green) with markers (red) of hepatocytes (HNF4α, nuclear) **(A)**, endothelial cells (CD31, cytoplasmic) **(B)**, biliary epithelial cells (CK19, cytoplasmic) **(C)**, HSCs (desmin, cytoplasmic) **(D)**, and

infiltrating macrophages (F4/80, cytoplasmic) **(E)** in normal and ALF mice (400×). n = 3 mice per group.

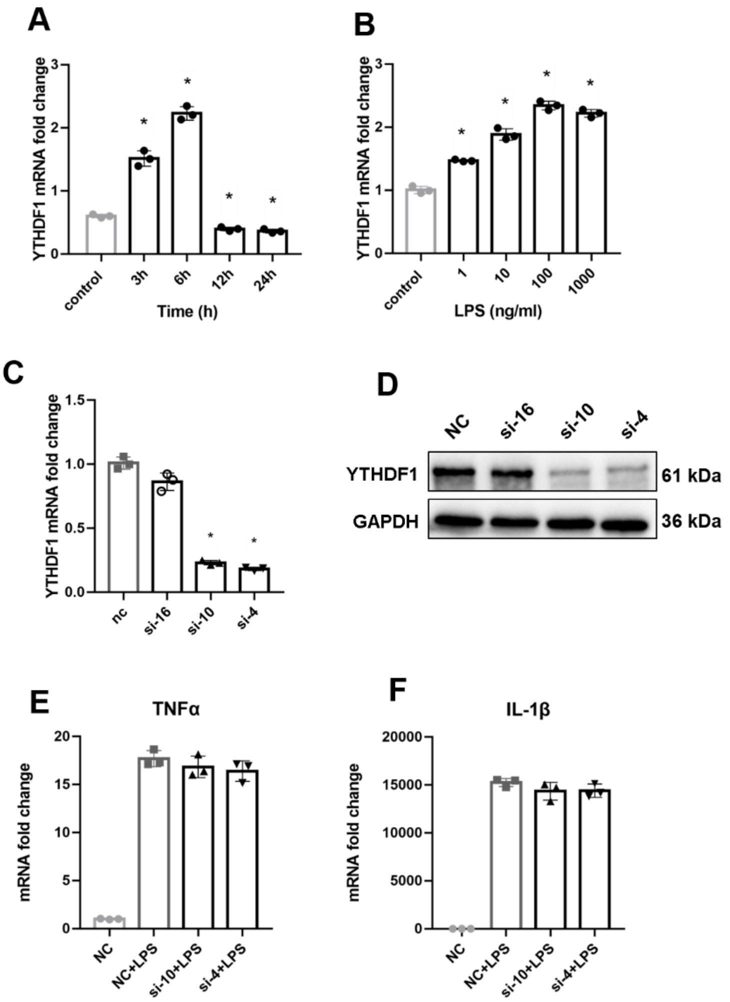

Figure S4. The effect of YTHDF1 on macrophage after stimulation with LPS. (A) Relative mRNA levels of YTHDF1 expression in RAW 264.7 cells treated with LPS (1000 ng/ mL) for different times (0-24 hours), n = 3 per group. (B) Relative mRNA levels of YTHDF1 expression in RAW 264.7 cells treated with LPS (0-1000 ng/ mL) for 6h. n = 3 per group. (C-D) qRT-PCR and Western blotting were used to verify the transfection efficiency of YTHDF1 knockdown in RAW 264.7 cells. (E) The expression levels of TNF-α and IL-1β in RAW 264.7 cells transfected with

siYTHDF1 or NC stimulated with 100 ng/ mL LPS for 6 h, n = 3 per group. The data are shown as mean ± S.D. $^{*}P < 0.05$.

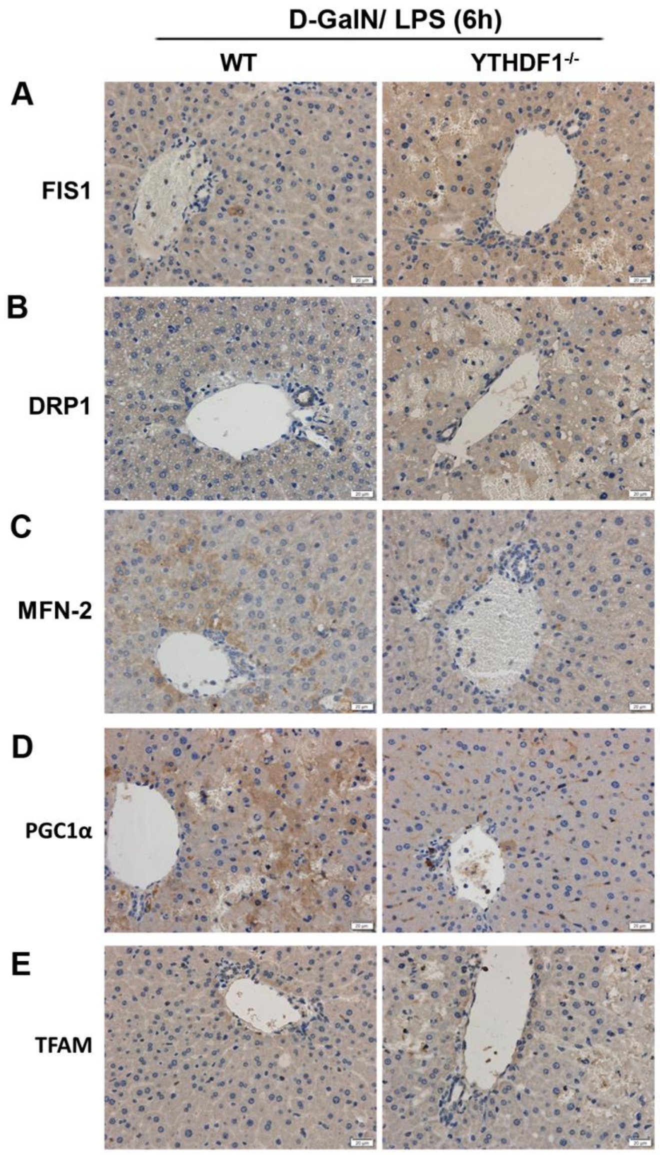

Figure S5. Representative images of IHC staining of mitochondrial fission and fusion related proteins in the livers of WT mice and YTHDF1$^{-/-}$ mice 6 h after D-GalN/LPS insult. Representative photos of FIS1(A)，DRP1 (B), MFN-2 (C), PGC1α (D) and TFAM (E) staining (400×)

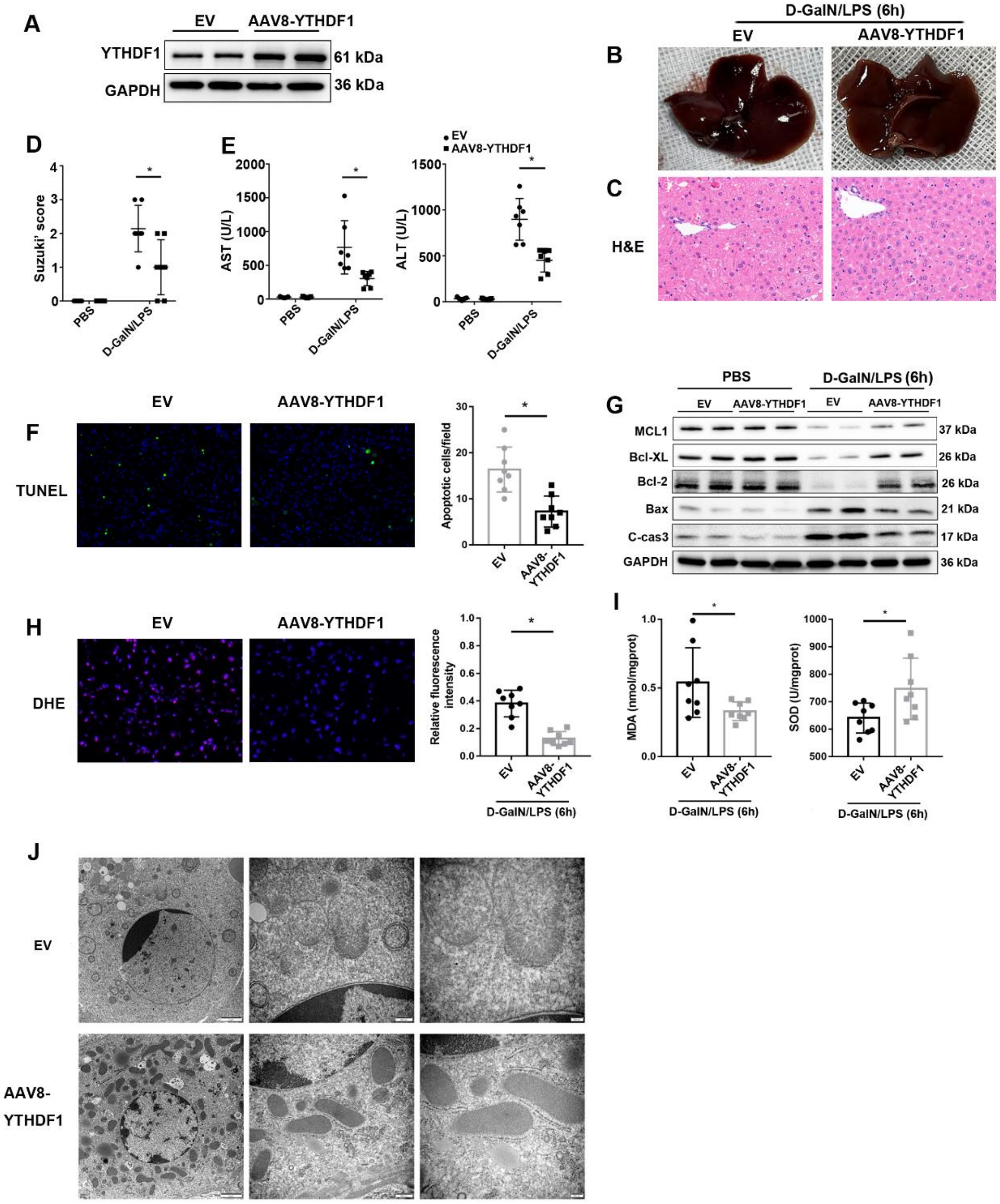

Figure S6. Hepatocyte-specific YTHDF1 overexpression protected mice against D-GalN/LPS-induced ALF. An ALF model induced by D-GalN/LPS insult was established 4 weeks following AAV8-YTHDF1 or empty vector (EV) injection. **(A)** Western blot analysis was used to verify the overexpression of YTHDF1 in the livers, n=4 per group. **(B, C)** Representative images of gross liver morphology **(B)** and H&E

staining **(C)** from AAV8- YTHDF1 and EV mice subjected to D-GalN/LPS treatment (400×, n=4 per group). **(D, E)** Suzuki scores of liver sections **(D)** and Serum ALT/AST activity **(E)** in AAV8- YTHDF1 and EV groups 6 h after D-GalN/LPS treatment (n=7 per group). **(F)** Representative images of TUNEL staining in the liver lobes of AAV8-YTHDF1 and EV mice 6 h after D-GalN/LPS treatment (400×; n=4 per group). **(G)** The protein levels of cell death-related genes in the livers of mice from the indicated groups after D-GalN/LPS insult (n=4 per group). **(H)** Representative images of DHE fluorescence staining in the livers from AAV8- YTHDF1 and EV mice (400×, n=4 per group). **(I)** MDA and SOD levels (n=7 per group). **(J)** Electron microscopy images showing ultrastructural alterations of the livers from the indicated mouse groups.

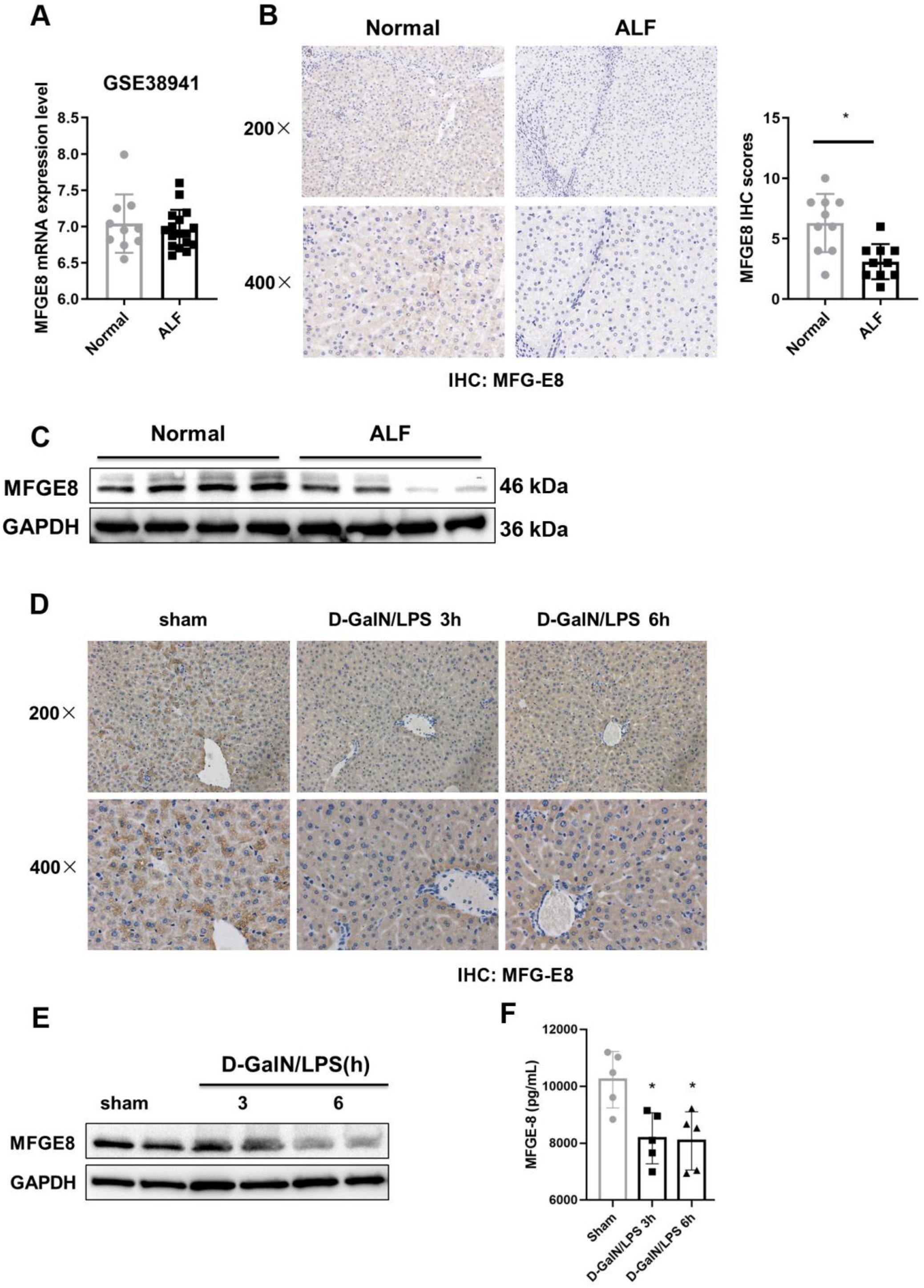

Figure S7. MFG-E8 expression downregulation is associated with ALF. (A) mRNA levels of MFG-E8 in ALF patients according to a GEO database (GSE38941). **(B)** Representative images of MFG-E8 expression in the explanted livers of patients with ALF and normal livers from patients with hepatic hemangioma. (n = 10 subjects per

group, 24 images per subject). Scale bar, 20 µm. For statistical analysis, two-tailed Student's t-test was used. * indicates $P<0.05$ compared with the controls. **(C)** Western blotting (n =4) showing MFG-E8 protein expression in the livers of the ALF and control group. GAPDH served as a loading control. n = 4 individuals per group. **(D)** Representative images of IHC staining showing MFG-E8 expression profiles in the livers of mice after D-GalN/LPS treatment for the indicated periods. n = 4 mice per group, 24 images per mouse. **(E)** MFG-E8 protein expression levels in the livers of mice subjected to D-GalN/LPS for the indicated periods. n = 4 mice in each group. **(F)** The serum MFG-E8 levels of mice subjected to D-GalN/LPS for the indicated periods. n = 5 mice in each group.

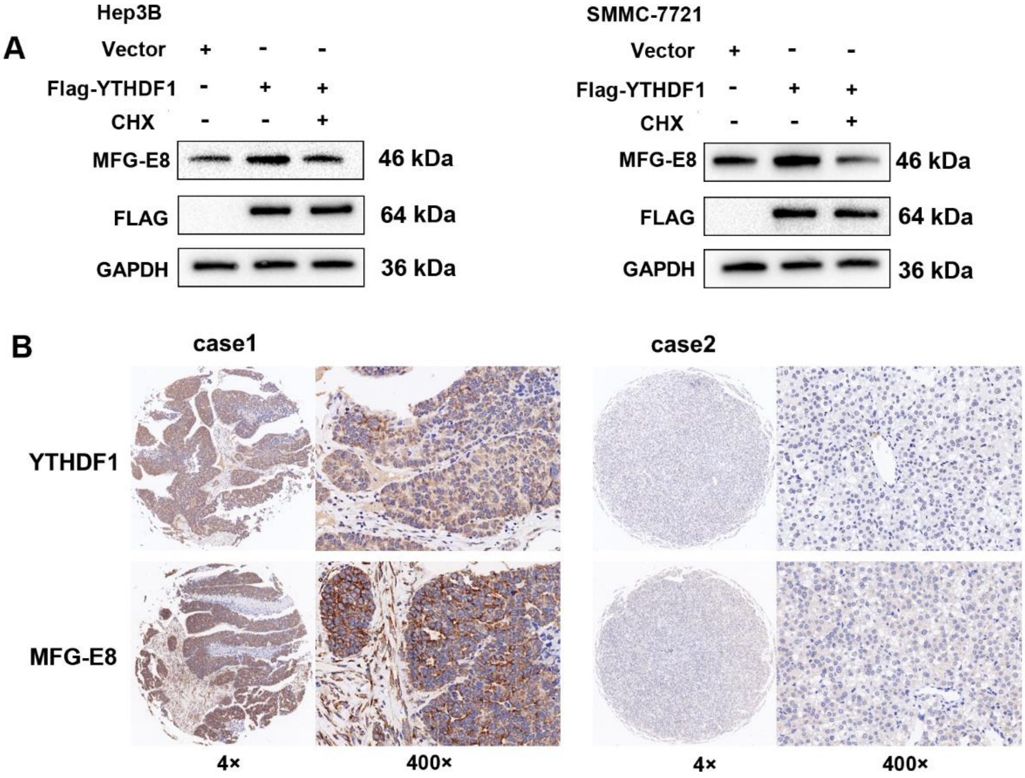

C

Expression	YTHDF1		
**MFG-E8**	low	high	
low	34	11	*P*=0.015
high	28	26	

Figure S8. YTHDF1 regulated MFG-E8 translation. (A) Western blot analysis of MFG-E8 protein expression upon overexpression of YTHDF1 and/or CHX treatment in liver cells. **(B)** Tissue sections from HCC patients were subjected to IHC staining for YTHDF1 and MFG-E8. **(C)** Correlation between YTHDF1 expression and MFG-E8 expression in HCC tissues.

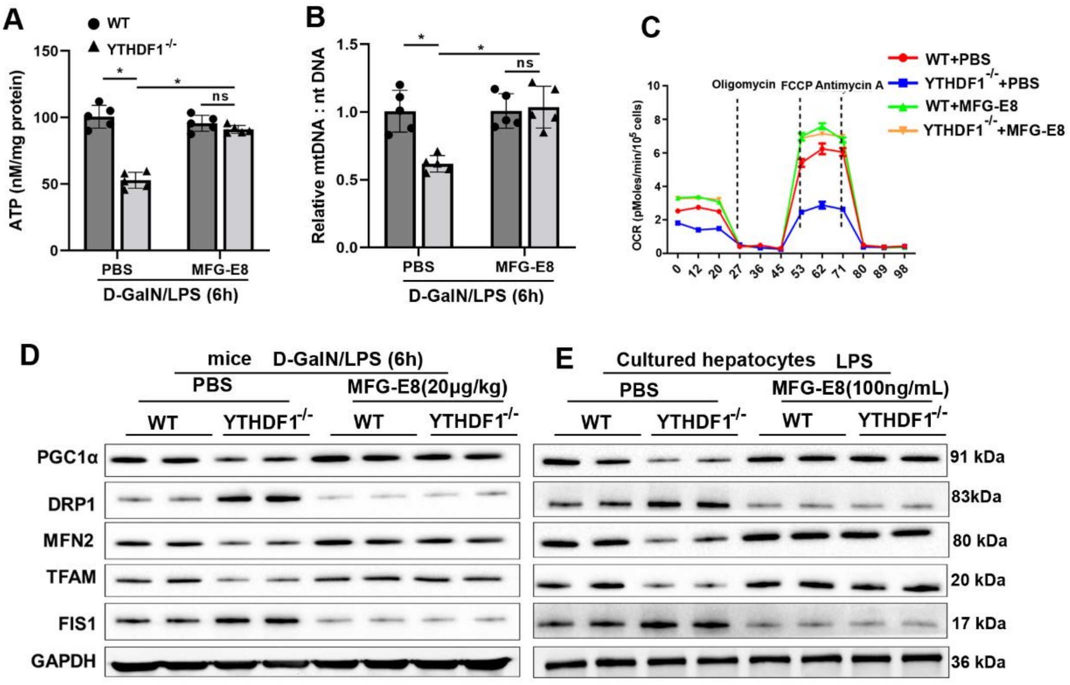

Figure S9. MFG-E8 administration reverses the mitochondrial damage of YTHDF1 knockdown on hepatocytes (A) ATP level in the liver tissues (n=5); **(B)** Relative mitochondrial DNA (mtDNA) copy number (mtDNA-to-nDNA) (n=5). **(C)** Representative result of respiration in WT and YTHDF1$^{-/-}$ mouse primary hepatocytes treated with recombinant MFG-E8 or PBS after LPS stimulation; **n = 3. (D)** Protein expression of PGC-1α, Tfam, Fis-1, Mfn-2 and Drp-1 in the livers in YTHDF1$^{-/-}$ and WT mice subjected to recombinant MFG-E8 or vehicle injection 2 h before D-GalN/LPS insult (n=2 per group). **(E)** PGC-1α, Tfam, Fis-1, Mfn-2 and Drp-1 protein expression in isolated hepatocytes of YTHDF1$^{-/-}$ and WT mice treated with recombinant MFG-E8 or vehicle was analyzed by Western blotting (n=2 per group). The data are shown as the mean ± S.D.; $^{*}P < 0.05$; ns indicates no significance.

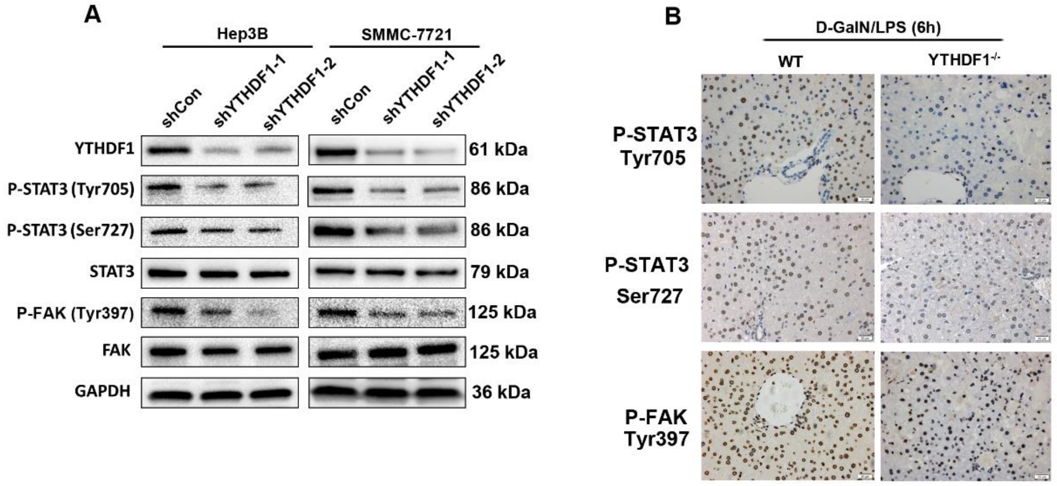

Figure S10. YTHDF1 modulated MFG-E8- FAK-STAT3 signaling in ALF. (A) Western blot analysis of the expression of FAK and STAT3 in shYTHDF1 or shControl HCC cells. **(B)** Representative photos of p-STAT3 and p-FAK staining (400×) in YTHDF1$^{-/-}$ and WT mice (400×).

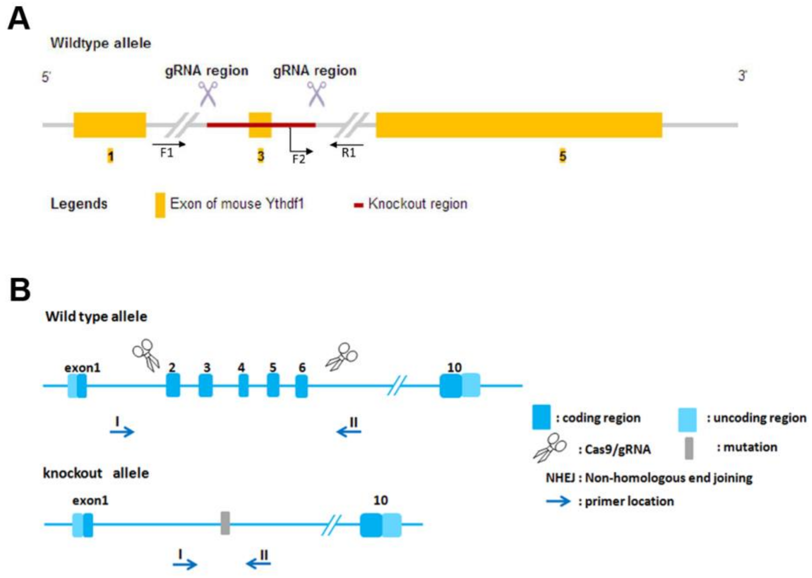

Figure S11. Generation of knockout mice. (A) Strategy used to knock out the YTHDF1 gene. **(B)** Strategy used to knock out the MFG-E8 gene.

Supplementary Tables

Table S1 Primers used in the study

**Primers used in RT-qPCR**		
name	sequence	(5’-3’)
m-GAPDH	F:	CATCACTGCCACCCAGAAGACTG
	R:	ATGCCAGTGAGCTTCCCGTTCAG
m-METTL3	F:	CAGTGCTACAGGATGACGGCTT
	R:	CCGTCCTAATGATGCGCTGCAG
m-METTL14	F:	AGAGTGCGGATAGCATTGGTGC
	R:	CTCCTTCATCCAGACACTTCCG
m-WTAP	F:	AGTGCCTGGAAGTTTACGCCTG
	R:	GCTTCAAGCTGTGCAATACGGC
m-VIRMA	F:	CCACTGCCTTACGTGTTCTCTG
	R:	AGGTGTCCATTCCTTCAGCAGAA
m-RBM15	F:	TCCTGACCGAAGTCCAGAACTG
	R:	TCTGGCTCTTCTCCAAACTGCC
m-RBM15b	F:	GGCAAGTTAGAGGAAGAACACAT
	R:	GAGAAGGTAGGTGGAGAAGGA
m-ZC3H13	F:	GGAGCATTCTCCTGACAGTGAC
	R:	CCTTGCCTATCTTCTGTGAGGTG
m-ALKBH5	F:	TCGGAACCTGTGCTTTCTCTGC
	R:	CTTCCTGAGAATGATGACCGCC
m-FTO	F:	GCCTCGGTTTAGTTCCACTCAC
	R:	GTCGCCATCGTCTGAGTCATTG
m-YTHDF1	F:	GCATCAGAAGGATGCAGTTCATG
	R:	GATGGTGGATAGTAACTGGACAG
m-YTHDF2	F:	GGTTCTGTGCATCAAAAGGATGG
	R:	CCAAAGAATAGGAAAAGCCAATGG
m-YTHDF3	F:	GGTTCGATTCATCAAAAAGATGCTG
	R:	GATCTGACATTGGTGGATAGCTG
m-YTHDC1	F:	CCAAGTCTCCTACACCAGATGG
	R:	AGAACCACTGCTTCCTGTCTCG
m-YTHDC2	F:	CCTGTTACTGTCCTGGTGTTC
	R:	CTCACTGTCACTGCTGTCAT
m-RBMX	F:	CTCTGCCAGTAAAACGAGGACC
	R:	CTCCGTAACCATCTCTTCCACG
m-HNRNPC	F:	GCTCTGTGCATAAAGGCTTTGCC
	R:	CTCGGTTCACTTTTGGCTCTGC
m-IGF2BP1	F:	CCTGGCTCATAACAACTTCGTCG
	R:	CCTTCACAGTGATGGTCCTCTC
m-IGF2BP2	F:	CCACCCAGTTTGTTGGAGCCAT
	R:	GGATAGTAATGGACTTCTCCGCG
m-IGF2BP3	F:	CCACCCAGTTTGTTGGAGCCAT
	R:	GGATAGTAATGGACTTCTCCGCG
m-MFGE-8	F:	GAGCAACAGTGCCAAGGAATGG
	R:	ACTGTGGGCTACCTTGTAGGAC
m-TNFA	F:	GGTGCCTATGTCTCAGCCTCTT
	R:	GCCATAGAACTGATGAGAGGGAG
m-IL 1β	F:	TGGACCTTCCAGGATGAGGACA
	R:	GTTCATCTCGGAGCCTGTAGTG

h-YTHDF1	F:	CAAGCACACAACCTCCATCTTCG
	R:	GTAAGAAACTGGTTCGCCCTCAT
h-MFGE-8	F:	CTGGACAAGCAGGGCAACTTCA
	R:	TGATGCCTGTCACCTCCTTCGA
**Primers used in Genotyping**		
name	sequence	(5>3)
YTHDF1	F1	5'-CTGACTGGTCCTCTGTTGCTAGG-3'
	R1	5'-GTCCAAGAGACCCTGCATCACTG-3'
	F2	5'-CATGGAGATTGGGTACAGGCAGAC-3'
MFG-E8	F1	ATGTATTTGCTTTATTGTCTA
	R1	ACGGTGCCCACTTTGTTA
	F2	ATTTTCTAGTCCCTTGTCATCAGC
	R2	TAAAAACAAAACAAAACGCAAACA
**shRNA target oligo**		
shYTHDF1-1		GATACAGTTCATGACAATGA
shYTHDF1-2		GAAACGTCCAGCCTAATTCT
shMFGE-8-1		GTGGGTAACTGGAACAAA
shMFGE-8-2		GGACACGAATTCGATTTCA
**siYTHDF1 sequences for transcription (5'-3')**		
siRNA-10		CCAUCCAUUGGAUUUCCUUTT
		AAGGAAAUCCAAUGGAUGGTT
siRNA-4		GGACAUUGGUACUUGGGAUTT
		AUCCCAAGUACCAAUGUCCTT
**gRNA target sequence**		
YTHDF1	gRNA1	GTTCGGTCCCACCTTGGGGC GGG
	gRNA2	GACGGCAAGTCTCCTGATGC AGG
MFG-E8	gRNA1	TCCTTAGACATACCAGAAGA GGG
	gRNA2	CCCTGCCCTGGAAGCGGTGG TGG

Table S2 Primary antibodies for WB, IHC and IF.

Antibody	Assay Type	Company
YTHDF1 (mouse)	WB;IHC	Abcam, ab252346
YTHDF1 (human)	WB;IHC;IF	Proteintech, 17479-1-AP
MFG-E8	WB;IHC;IF	SANTA CRUZ Biotechnology, sc-271574
CD31 (mouse)	IF	Abcam, ab281583
F4/80 (mouse)	IF	Abcam, ab6640
CK19 (mouse)	IF	Abcam, ab52625
Desmin (mouse)	IF	Abcam, ab32362
HNF4a (mouse)	IF	Abcam, ab201460
GAPDH	WB	Abcam, ab8245
FLAG	WB	Sigma-Aldrich, F1804
Myc-Tag	WB	Cell Signaling Technology, 2276
Bcl-2	WB	Abcam, ab182858
Bax	WB	Abcam, ab32503
C-cas3	WB	Cell Signaling Technology, 9664
FIS1 (mouse)	WB;IHC	Genetex, GTX111010
DRP1 (mouse)	WB;IHC	Abcam, ab184247
TFAM (mouse)	WB;IHC	Genetex, GTX103231
PGC1α（mouse）	WB;IHC	Genetex, GTX31921
MFN-2 (mouse)	WB;IHC	Abcam, ab124773
Phospho-FAK (Tyr397)	WB;IHC	Cell Signaling Technology, 3283
FAK	WB	Cell Signaling Technology, 3285
Phospho-STAT3 (Tyr705)	WB;IHC	Cell Signaling Technology, 9145
Phospho-STAT3 (S727)	WB;IHC	Abcam, ab32143
STAT3	WB	Cell Signaling Technology, 9139
Bcl-XL	WB	Abcam, ab32370
MCL1	WB	Abcam, ab32087
HRP-conjugated Affinipure Goat Anti-Mouse IgG(H+L)	WB	Proteintech, SA00001-1
HRP-conjugated Affinipure Goat Anti-Rabbit IgG(H+L)	WB	Proteintech, SA00001-2

Materials and Methods

Bioinformatics analysis

Hepatitis B virus (HBV)-associated ALF mRNA expression profile data (GSE38941) (ALF samples=4, Normal samples=10), murine model of primary mitochondrial dysfunction mRNA expression profile data (GSE10904) (Primary mitochondrial dysfunction samples=3, Normal samples=3) were downloaded from GEO database. Then the data were analyzed by R (V3.3, http://www.bioconductor.org) with edgeR package.

Cell culture

Hep3B cells and SMMC-7721 cells were purchased from American Type Culture Collection (ATCC, Manassas, VA, USA), and cultured in DMEM or RPMI 1640 (Biological Industries) supplemented with 10% FBS (Biological Industries) and 1% penicillin/streptomycin (Beyotime Biotechnology,) at 37˚C in a humidified chamber with 5% CO_2.

Quantitative real-time PCR analysis

Total RNA was isolated using Trizol reagent (Invitrogen, California, USA). Then the RNA was reverse-transcribed into cDNA by using PrimeScript™ RT Master Mix (Takara, Japan). Further the real-time PCR assay was conducted by using TB Green® Premix Ex Taq™ II (Takara, Japan). Quantitative-PCR was performed using the ABI StepOne Plus Detection System. The results were normalized to GAPDH for mRNA expression measurement. The sequences of primer used were shown in Supplementary Table 1.

RNA m^{6}A Quantification

Total RNA was isolated using TRIzol (Invitrogen, CA, USA) and further treated with deoxyribonuclease I (Sigma-Aldrich, St. Louis, USA). The m^{6}A level in mRNA was measured by EpiQuik m^{6}A RNA Methylation Quantification Kit (Epigentek, NY, USA) following the manufacturer's instruction. Briefly, 200 ng RNAs were coated on wells. Capture antibody solution and detection antibody solution were then added to wells separately. The m^{6}A levels colorimetrically quantified by reading the absorbance at a wavelength of 450 nm, and then calculations were performed based on the standard curve.

RNA m^{6}A dot blots

Dot blots were performed as previously described [1]. Isolating total RNA first and then the RNAs (200ng) were spotted onto a nylon membrane (Sigma-Aldrich, GERPN1210B). The membranes were then UV cross-linked, blocked, incubated with m^{6}A antibody (Abcam, ab284130) and HRP-conjugate anti-rabbit IgG and finally detected with a DAB peroxidase substrate kit. The same amount RNAs were spotted on the membrane, stained with 0.02% methylene blue in 0.3 M sodium acetate (pH 5.2) for 2 h and washed with RNase-free water for 5 h.

Histopathological analysis and assessment of apoptosis

Liver tissues were fixed in formalin and embedded in paraffin wax, and sections in 5 μm were stained with H&E using a standard protocol, and then analyzed by light microscopy. Hepatocyte apoptosis in liver tissue was observed by the terminal deoxynucleotidyl transferase dUTP-mediated nick-end labeling (TUNEL) assay using

a commercially-available kit (Roche, USA).

Serum aminotransferase activities

Plasma samples were taken from the mice 6 h after D-GalN/LPS injection. Serum levels of ALT, AST were measured using the the commercially available kits (Nanjing Jiancheng Bioengineering Institute, Nanjing, China).

Determination of hepatic lipid peroxidation and antioxidant enzymes

Liver tissue was homogenized in cold 50mM Tris HCl (pH 7.5, 4 ml per g of tissue) with a glass-Teflon tissue homogenizer, and centrifuged at 1000 g for 15 min at 4 °C. Lipid peroxidation was assessed by detecting malondialdehyde (MDA), an end product of lipid peroxidation. Hepatic MDA was measured using a thiobarbituric acid method as previously described. Moreover, superoxide dismutase (SOD) and glutathione (GSH) in the supernatants were immediately detected using the commercially available kits (Nanjing Jiancheng Bioengineering Research Institute,
Nanjing, China).

Determination of reactive oxygen species (ROS)

A fluorescent probe named Dihydroethidine hydrochloride (DHE; Sigma-Aldrich, St. Louis, USA) was used to detect ROS generation. Each tissue section was incubated with 5 μM DHE in a light-protected humidified chamber at 37 °C for 30 min. The fluorescence intensity was detected.

Western blotting and antibodies

Liver tissues or cells were lysed in RIPA buffer supplemented with Protease inhibitor cocktail (bimake, USA). Protein samples were separated by SDS-PAGE and

transferred onto PVDF membrane (#162-0177; Bio-Rad). The membrane was blocked with 5% defatted milk in PBST (0.1% Tween-20 in PBS) and incubated overnight with the indicated primary antibodies. After extensive washing with PBST, the membrane was incubated with HRP-conjugated secondary antibody. The chemiluminescence detection procedure was performed using ECL western blotting substrate. The primary antibodies used in this study were shown in Supplementary Table 2.

Detection of mtDNA/nDNA Ratios

Total DNA was isolated with a commercial kit (69504, Qiagen, Valencia, CA) following the instructions. To quantify the mtDNA/nDNA ratios, qPCR was used to amplify mtATP6 gene (from the mitochondrial genome) and Rpl13a gene (from the nuclear genome), and the protocols were performed as previously described [2]. The mtDNA/nDNA ratios was calculated by a comparative Ct method, using the following equation: mtDNA/nDNA $= 2^{-\Delta Ct}$.

Establishment of YTHDF1 or MFG-E8 knocking down stable liver cell lines

YTHDF1 or MFG-E8 shRNA knockingdown lentivirus was purchased from genepharma (Shanghai, China). Cells were seeded in six-well plates for overnight and further infected with virus and polybrene. Positive clones were selected by using puromycin for following 14 days to obtain the stable cell lines.

RNA sequencing (RNA-seq)

RNA-seq was processed according to the instructions of NEBNext Ultra RNA Library Prep Kit for Illumina (New England BioLabs). Briefly, total RNAs were isolated from YTHDF1-knocking down or control cells using Trizol reagent. Poly(A)

RNA was subsequently purified by using PolyTtract mRNA Isolation System and used to generate cDNA libraries. All samples were sequenced on Illumina Novaseq™ 6000 platform. Sequence reads were mapped to the human genome version hg38 by using Illumina sequence analysis pipeline.

m^6A-seq

Total RNAs were extracted by TRizol (ThermoFisher, USA) from stable shYTHDF1 Hep3B cells and the controls. Polyadenylated RNA was further enriched from total RNA using the Dynabeads mRNA Purification Kit (Invitrogen). RNA samples were fragmented into~100-nucleotide-long fragments by sonication. Fragmented RNA (100 ng mRNA or 5 μg total RNA) was used for m6A immunoprecipitation (m6A-IP) with the EpiMark N6-methyladenosine enrichment kit (NEB E1610S) according to the manufacturer's protocol. RNA was enriched through RNA Clean & Concentration-5 (Zymo Research) and used for library generation with SMARTer Stranded Total RNA-Seq Kit (Takara). Sequencing was performed at the University of Chicago Genomics Facility on an Illumina Novaseq™ 6000 machine in single-read mode with 50 bp per read. Sequencing reads were aligned to the genome GRCh38 (hg38) by STAR (version 2.6.0c) 30. The m6A-enriched regions (peaks) in each m6A-IP sample were detected by MACS2 (version 2.1.1.20160309)31 with $q < 0.01$ and the corresponding m6A-input sample was used as the control. Peaks that were detected by both replicates were considered as high-confidence peaks. The peak annotation and binding motif were analyzed by HOMER (version 4.9).

RIP-seq

Cells were washed twice with PBS, collected and then the pellet was resuspended in IP lysis buffer (150mM KCl, 25mM Tris (pH 7.4), 5mM EDTA, 0.5mM DTT, 0.5% NP40, 1× protease inhibitor, 1 U/μL Nase inhibitor). The lysate was harvested by centrifugation at 12 000 g for 10 min after incubation for 30 min. Antibodies and 40 μL of protein G beads (Invitrogen, USA) were added into the lysate followed by incubation overnight at 4°C. After washed three times with wash buffer (150mM KCl, 25mM Tris (pH 7.4), 5mM EDTA, 0.5mM DTT, 0.5% NP40), co-precipitated RNAs were extracted by Trizol reagent, ethanol-precipitated with glycogen (Invitrogen, USA). The enrichment of RNAs was normalized to IgG. For sequencing, rRNAs was depleted by using the NEBNext rRNA depletion kit (New England BioLabs). cDNA libraries were produced by employing NEBNext UltraRNALibrary Prep Kit for Illumina (New England BioLabs) and sequenced on Illumina HiSeq X Ten platform.

m^{6}A-RT-qPCR

Total RNA were extracted using Trizol, followed by mRNA purified using the GenElute mRNA Miniprep Kit (Sigma). Then 5 μg mRNA was fragmented to 200-300 nt in length. After saving 0.5 μg of the fragmented mRNA as input, the remaining mRNA was used for m^{6}A immunoprecipitation with 5 μg m^{6}A antibody or mouse IgG in 500 μl of IP buffer to obtain m^{6}A pull down portion. m^{6}A IP RNAs were analyzed by quantitative RT-PCR.

RIP-RT-qPCR

YTHDF1 immunoprecipitation assay was performed with a Magna RIP™ RNA-Binding Protein Immunoprecipitation Kit (Millipore). Briefly, cell pellet was re-

suspended and lysed in RIP lysis buffer. 50 μl protein A/G magnetic beads were incubated with 5 μg anti-YTHDF1 antibody in RIP wash buffer at room temperature for 30 min. Then, lysates were immunoprecipitated with beads-antibody complex in RIP immunoprecipitation buffer at 4 ℃ for 4 hours before 10% of RIP lysates was removed for input. Normal Rabbit IgG was used as a negative control (Millipore). The magnetic bead-bound complexes were centrifuged and washed for 5 times. The precipitated RNA samples were extracted and purified with Phenol: chloroform: isoamyl alcohol (125:24:1), and the immunoprecipitated RNA was then analyzed by qPCR.

Apoptosis assay

Apoptotic cells were detected by Annexin V-FITC/PI Apoptosis Detection Kit (BD, USA) following the manufacturer's recommendations. The cells were analyzed with a flow cytometry (ACEA Biosciences, Inc.). The sum of early and late apoptotic cell percentage was defined as apoptotic cell percentage.

Transmission electron microscopy

Seventy nm ultra-thin sections of liver tissue samples were stained with uranyl acetate and lead citrate. Hepatic ultrastructure was evaluated using a transmission electron microscope (HT7700, Hitachi, Japan) by a single electron microscopist.

Enzyme-linked immunosorbent assay (ELISA)

Serum MFG-E8 levels were measured using mouse MFG-E8 Quantikine ELISA Kit (MFG-E80) from RD System, Inc. Minnesota, USA. LDH levels were measured using corresponding assay kits (SEB864Mu) from Cloud-Clone Corp USCN Life Science, Wuhan, China. The procedures were performed according to the

manufacturer’s instruction.

Immunofluorescence staining

The procedure of immunofluorescence staining in the cells was performed as previous described [3]. The primary antibody rabbit anti-MFG-E8 antibody was incubated overnight at 4°C. Then the second antibody goat anti-mouse antibody were incubated 50 min at room temperature and counterstained with 4’-6-diamidino-2-phenylindole (DAPI). The sections were observed under a Nikon Eclipse Ti confocal microscope, and representative fields were chosen for application.

ATP content

ATP was detected using the ATP Assay Kit (S0026, Beyotime, China), and the ATP content was normalized to total protein with a unit of nmol per milligram protein (nmol/mg protein).

Mitochondrial respiration assay

Oxygen Comsumption Rate (OCR) was determined using the XF24 Extracellular Flux Analyzer Seahorse Bioscience). Briefly, 2 $\times 10^5$ primary hepatocytes were seeded onto 24-well plates and incubated overnight. Then, cells were washed with Seahorse buffer. Cell Mito Stress Test Kit was used to measure cellular mitochondrial function, 2 μg/ml oligomycin, 0.5 μM FCCP, and 1μM rotenone was automatically injected to determine the OCR, according to the manufacturer's instructions. OCR values were normalized to cell numbers. Data were from triplicated experiments and were plotted as mean ± S.D.

Protein stability

To evaluate protein stability, cells were treated with CHX (100 μg/ml) during indicated times and harvested. Then protein expression of MFG-E8 was determined by western blot analysis.

Hepatocyte isolation and culture

Hepatocytes were isolated as previously described [4]. Further, the hepatocytes were cultured in DMEM supplemented with 10% fetal bovine serum and 1% penicillin-streptomycin in a 5% CO2 water-saturated incubator at 37 °C. Primary hepatocytes were treated with 20 ng/ml of TNF for 6 hours after 16 hours of culture.

Cell viability and cell cytotoxicity assays

Cell viability was assessed by the Cell Counting Kit-8 (CCK8) Assay Kit (Dojindo). Cell cytotoxicity was assessed by measuring lactate dehydrogenase release from the hepatocytes into the medium and was detected by using a Lactate Dehydrogenase Assay kit (ab102526, abcam).

References

1. Song H, Feng X, Zhang H, Luo Y, Huang J, Lin M, et al. METTL3 and ALKBH5 oppositely regulate m(6)A modification of TFEB mRNA, which dictates the fate of hypoxia/reoxygenation-treated cardiomyocytes. Autophagy. 2019; 15: 1419-37.
2. Zhang Y, Yao Y, Qiu X, Wang G, Hu Z, Chen S, et al. Listeria hijacks host mitophagy through a novel mitophagy receptor to evade killing. Nat Immunol. 2019; 20: 433-46.
3. Dong J, Ke MY, Wu XN, Ding HF, Zhang LN, Ma F, et al. SRY is a Key Mediator of Sexual Dimorphism in Hepatic Ischemia/Reperfusion Injury. Annals of surgery. 2022; 276: 345-56.
4. Lan T, Hu Y, Hu F, Li H, Chen Y, Zhang J, et al. Hepatocyte glutathione S-transferase mu 2 prevents non-alcoholic steatohepatitis by suppressing ASK1 signaling. Journal of hepatology. 2022; 76: 407-19.
